# The phenotypic and demographic response to the combination of copper and thermal stressors strongly varies within the ciliate species, *Tetrahymena thermophila*

**DOI:** 10.1111/1758-2229.13307

**Published:** 2024-09-30

**Authors:** Doufoungognon Carine Estelle Koné, Staffan Jacob, Michèle Huet, Hervé Philippe, Delphine Legrand

**Affiliations:** ^1^ Centre National de la Recherche Scientifique Station d'Ecologie Théorique et Expérimentale, UAR2029 Moulis Ariège France

## Abstract

Copper pollution can alter biological and trophic functions. Organisms can utilise different tolerance strategies, including accumulation mechanisms (intracellular vacuoles, external chelation, etc.) to maintain themselves in copper‐polluted environments. Accumulation mechanisms can influence the expression of other phenotypic traits, allowing organisms to deal with copper stress. Whether copper effects on accumulation strategies interact with other environmental stressors such as temperature and how this may differ within species are still unsolved questions. Here, we tested experimentally whether the combined effect of copper and temperature modulates traits linked to demography, morphology, movement and accumulation in six strains of the ciliate *Tetrahymena thermophila*. We also explored whether copper accumulation might modulate environmental copper concentration effects on phenotypic and demographic traits. Results showed high intraspecific variability in the phenotypic and demographic response to copper, with interactive effects between temperature and copper. In addition, they suggested an attenuation effect of copper accumulation on the sensitivity of traits to copper, but with great variation between strains, temperatures and copper concentrations. Diversity of responses among strains and their thermal dependencies pleads for the integration of intraspecific variability and multiple stressors approaches in ecotoxicological studies, thus improving the reliability of assessments of the effects of pollutants on biodiversity.

## Graphical abstract

The combined effects of copper and temperature induced unexpected responses across the species, *Tetrahymena thermophila*. Copper had harmful effects on demography; however, its accumulation mitigated these effects. Temperature modulated the response of strains. Intraspecific variability is crucial for understanding the effects of heavy metals on biodiversity.

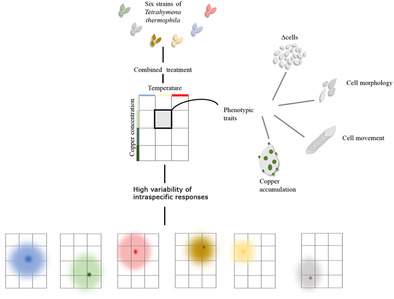

## INTRODUCTION

Some heavy metals are essential elements of proteins' structure and are required for organism's metabolism and cell growth (da Silva & Williams, 1992). However, if their concentration becomes too high in the environment in a bioavailable form, they can become toxic (Graham, 1999; Jeyakumar et al., 2023; Nriagu & Pacyna, 1988). The presence of heavy metals in aquatic environments is a major concern today due to water discharges from anthropogenic activities such as agricultural run‐off, industrial activities, exploitation mining and household wastes (e.g., Kapoor & Singh, 2021; Karbassi et al., 2006; Verma & Dwivedi, 2013; Zamora‐Ledezma et al., 2021). Unlike many persistent organic pollutants, metals cannot be degraded. Thus, they accumulate across organisms, disturbing the natural ecological balance (Kong et al., 1995; Luoma & Rainbow, 2005; Naimo, 1995), in turn impacting ecosystems' functioning and human health (Aziz et al., 2023; Briffa et al., 2020; Jaiswal et al., 2018). As a result, many heavy metals are considered a priority among environmental pollutants (Keith & Telliard, 1979; Zait et al., 2022).

To resist heavy metal pollution, organisms set up various tolerance strategies. They include external accumulation or biosorption (Balíková et al., 2022; Gupta and Diwan, 2017; Mohite et al., 2017; Sedlakova‐Kadukova et al., 2019; Sulaymon, 2014), and internal accumulation or bioaccumulation. Biosorption refers to cell surface sorption mechanisms based on physicochemical interactions between the heavy metal and the functional groups of the cell membrane. It consists mainly of polysaccharides, lipids and proteins, which can bind to metals through various mechanisms (Balíková et al., 2022). Bioaccumulation is an intracellular accumulation of heavy metals involving the binding of heavy metals to intracellular compounds, specific enzymes and proteins such as metallothioneins (Bhardwaj et al., 2021; Mathivanan et al., 2021; Wang et al., 2014), which bind to metal ions in order to reduce their toxicities and protect cell damage. External and internal accumulation processes can occur simultaneously to reduce the toxicity of metal ions (Kaduková & Virčíková, 2005; Velásquez & Dussan, 2009). Accumulation mechanisms have been explored in a wide variety of organisms, like fishes (e.g., Jamil Emon et al., 2023; Panigrahi et al., 2021), molluscs (e.g., Naimo, 1995), crustaceans (e.g., Kouba et al., 2010) and microorganisms (e.g., Balíková et al., 2022; Somasundaram et al., 2018). Microorganisms deserve a particular focus due to their crucial importance in heavy metal bioremediation processes (e.g. Balíková et al., 2022; Gupta & Diwan, 2017; Martín‐González et al., 2006; Velásquez & Dussan, 2009). During accumulation processes, microorganisms' phenotypic traits can be modified. For example, some studies showed that the accumulation of zinc and copper rounded ciliate cells' shape and slowed down swimming (Nicolau et al., 1999; Nilsson, 1981; Peng et al., 2019; Somasundaram et al., 2018).

The responses of organisms to environmental pollution may change depending on intrinsic factors linked directly to the individual, such as genetic factors, and on extrinsic factors linked to the environment, such as temperature (Medina et al., 2007; Olkova, 2021). Firstly, recent analyses have shown that the response to multiple stressors is often very different from the response to a single stressor due to interactive effects that could either amplify or reduce the effects of each stressor (e.g., Pirotta et al., 2022). For example, some studies have shown that temperature can modulate pollutant effects on organisms (Schiedek et al., 2007; Sokolova & Lannig, 2008; Wang et al., 2019). Secondly, responses to environmental changes can strongly vary at the intraspecific level (e.g., Bolnick et al., 2003; Yang et al., 2020). Therefore, it is important to integrate both genotypic and environmental variability to better understand organism's responses to metal pollution (e.g., Delnat et al., 2022; Petitjean et al., 2020, 2021). Such a multidimensional approach (intraspecific and environmental variation) could improve the conclusions and the relevant applications for preserving freshwater ecosystems. Thus, a key aspect of our understanding of the effects of exposure to heavy metals on organisms is to determine whether they vary within species and according to the presence of other stressors. To study this complex question, we have focused on the combined effects of copper and temperature on the ciliate *Tetrahymena thermophila*.

Copper is one of the heavy metals found in the highest quantities in freshwater ecosystems due to its intensive agricultural use as an anti‐fungicidal agent (Bouson et al., 2017; Eisler, 1998; Okocha & Adedeji, 2012). Copper has been the subject of many studies investigating its environmental consequences and its deleterious effects on ecological processes, such as changes in community composition or the disruption of trophic relationships (Förstner & Prosi, 1979; Gardham et al., 2015; Hossain & Rakkibu, 1999; Nor, 1987; Soldo & Behra, 2000). In recent years, various studies have focused on the effects of copper toxicity on ciliates of the genus *Tetrahymena* and on their accumulation mechanisms. These studies indicate that copper reduces or inhibits their growth and modifies other physiological traits such as phagocytosis and locomotion (Arcila et al., 2019; Martín‐González et al., 2006; Nicolau et al., 1999; Nilsson, 1981). Some studies revealed that a strain of *Tetrahymena farahensis* can accumulate up to 54.9% of the copper present in its environment (Zahid et al., 2018). This could be explained by the existence of several accumulation mechanisms in this genus. For instance, a strain of *Tetrahymena pyriformis* and a strain of *Tetrahymena pigmentosa* are able to accumulate copper by producing metallothioneins (Piccinni et al., 1987; Santovito et al., 2001). It remains, however, unexplored if and how temperature influences these ciliates' cellular responses to copper exposure and if it exhibits substantial variation in these responses that could be explained by different copper accumulation strategies coexisting within the same ciliate species.

We selected *T. thermophila* because it is one of the most commonly used ciliate species in ecotoxicological and ecological research (e.g., Arcila et al., 2019; Campana et al., 2022; Dayeh et al., 2005; Gallego et al., 2007; Jacob & Legrand, 2021; Nilsson, 1981). *T. thermophila* is a freshwater bacterivorous eukaryotic microorganism (Collins & Gorovsky, 2005), living in the eastern United States where it is patchily distributed in freshwater ponds and streams (Zufall et al., 2013). The short‐term effects of stressors can be easily studied in this species (e.g., De Francisco et al., 2018). *T. thermophila* microscopic size (~20–50 μm), short asexual generation time (~5 h in our culture conditions) and high densities (up to millions of cells in a few millilitres) facilitate the analysis of pollutants effects on cell viability (Gerhardt et al., 2010). As such, the species can serve as an early warning indicator of negative environmental impacts (Maurya and Pandey, 2020).

Here, we experimentally evaluate copper toxicity in six strains of *T. thermophila* under varying thermal conditions. We aimed to (i) estimate intraspecific variability in *T. thermophila* demography (one important aspect of fitness) to a copper gradient under different thermal conditions, (ii) quantify morphological, swimming and copper accumulation changes along the copper and temperature gradients and (iii) investigate whether copper accumulation might modulate environmental copper concentration effects on demography and phenotypic traits. To do so, the toxic effect of copper was measured under controlled laboratory conditions at three temperatures spanning the thermal niche of *T. thermophila*. In parallel, we looked at the effect of copper and thermal treatments on a series of phenotypic parameters, including cell size, cell elongation, and swimming velocity. We finally estimated the ability of cells to accumulate copper in all combinations of copper and temperature conditions to determine to what extent copper accumulation from the media by cells could impact the other phenotypic traits.

## EXPERIMENTAL PROCEDURES

### Strains and culture conditions

Six strains of *T. thermophila* were used in this study: D3, D8, D10, D16, D19 and D21. Ciliates possess two nuclear genomes: the diploid micronuclear genome, only transcriptionally active during sexual reproduction (germline), and the polyploid macronuclear genome, controlling somatic cell functions during vegetative growth. The macronuclear genomes of our strains were recently sequenced (Derelle et al., 2023) showing that D8, D19 and D21 are closely related and very similar to SB210, which was used to obtain the reference genome of *T. thermophila* (Eisen et al., 2006). The three other strains, D3, D10 and D16, have distinct macronuclear genomes, D3 and D10 being natural isolates, while D16 is the result of a cross between a SB210‐like relative and an unknown presumably natural isolate (Derelle et al., 2023). These six strains have six distinct genotypes. These strains are routinely cultured in proteose peptone yeast extract (PPYE) controlled axenic liquid growth medium (0.6% w/v aqueous solution of proteose peptone [difco], added to 0.06% w/v yeast extract) and maintained at a constant temperature of 23 ± 1°C in 2 mL within 24‐well plates with transfers every ~10 days. The manipulations of the strains were exclusively carried out under sterile conditions using a laminar flow hood.

### Copper and thermal stressors treatments

The exposure conditions (copper concentration and exposure time) were determined in a pilot experiment, showing that the maximum concentration tolerable by the majority of cells is around 350 mg/L in our controlled conditions (Figure S1). We fixed an exposure time of 96 h (4 days) because, starting from a small fraction of cells in stationary phase, all strains should have entered the exponential growth phase, but none should have reached the stationary phase under our control condition (~4–6 h generation time in the exponential phase at 23°C). In our pilot experiment, for this control condition, only two asexual generations were observed overall (1.94 ± 0.27 generations) after 96 h. This means that cells have finished their latency phase and just begun their exponential phase. In such a short number of generations, consistent differences in trait values across the replicates of a given clonal strains should result from phenotypic plasticity. Indeed, in two generations, the selection of de novo mutations seems unrealistic. Rather, phenotypic plasticity in response to copper and thermal variation can account for rapid and convergent phenotypic shifts between replicates of the same strain.

Cells were exposed to four copper concentrations (CuSO_4_ · 5H_2_O, Sigma): 0, 150, 275 and 350 mg/L (following a pilot experiment in which we determined the maximum tolerable concentration of our six strains; Figure S1), and three different temperatures: 15, 23 and 31°C. These three experimental temperatures were chosen as follows: a low temperature yet ensuring survival (15°C), the standard culture temperature (23°C) and a temperature close to the growth optimum (31°C; Figure S2). A stock solution of copper was prepared by dissolving the metal salt (CuSO_4_  5H_2_O) in ultrapure deionised distilled water at a concentration of 5 g/L. Then, the exposure concentrations were prepared by diluting the stock solution with PPYE. Three replicates per strain were carried out for each copper concentration. These replicates were created by inoculating 2.5 mL (~2.5 × 10^5^ cells) of 7‐day culture of each strain into 50 mL tubes (Falcon) previously filled with 10 mL of the adequate exposure medium. Then, three replicates per strain were placed in three climatic chambers fixed at 15, 23 and 31°C, resulting in 216 experimental populations (6 strains × 4 copper concentrations × 3 temperatures × 3 replicates; Figure S3).

### Assessment of demography, morphological and movement traits from video analysis

For each experimental population and for both initial and final experimental time (96 h), two 10 μL samples were pipetted and placed in multi‐chamber counting slides (Kima precision cell). Fifteen‐second videos were immediately made for each chamber under dark‐field macroscopy (Axio Zoom V16, Zeiss). These videos were processed to extract the number of cells, their morphology and their swimming velocity (see examples in Cayuela et al., 2022; Junker et al., 2021; Morel‐Journel et al., 2020) using the BEMOVI R package (Pennekamp et al., 2015). Cell size was defined as cell area; cell elongation was calculated as the ratio between the major axis and the minor axis of cell meaning that the higher the value, the more elongated the cell; and cell swimming velocity was calculated as the total distance travelled by the cells divided by the duration of the trajectory.

To measure the impact of treatments on *T. thermophila* demography (one important element of fitness), we calculated the changes in the number of cells (Δ_cells_) over 96 h, which combines the effects of both survival and growth:

$$\Delta_{\text{cells}}=\frac{\mathrm{Nb}\;96\;h–\mathrm{Nb}\;\text{inoculated}}{\mathrm{Nb}\;\text{inoculated}}.$$

Nb 96 h corresponds to the number of cells present in the experimental treatments at the end of the experiment; Nb inoculated corresponds to the number of cells inoculated at the beginning of the experiment.

### Measurement of copper accumulation traits

We estimated copper accumulation by measuring the amount of copper eliminated by cells from the medium for each population after 96 h of exposure. We followed the method of Martín‐González et al. (2006). The 216 experimental tubes were filtered (0.22 μm membrane) to remove all cells. Soluble metal concentrations were measured from these free of cells media using an inductively coupled plasma emission spectrometer (ICP‐AES) Sequential‐Multichannel (SPECTRO). The spectrometer (ICP‐AES) was calibrated using copper standard solutions, considered certified reference materials (analyses performed by the Fast Bio‐analyses of Trace Elements Platform, LEFE, Auzeville Tolosane, France). Copper concentration accumulated by cells (accumulation concentration) was determined as the difference between the initial copper concentration and the final copper concentration (after 96 h of incubation with the cells); and the percentage of accumulated copper (accumulation percentage) as the ratio between the accumulated copper concentration and the initial copper concentration. Cell‐free controls were performed for each copper concentrations and for each temperature (Figure S4). The objective of these controls was to better calibrate the accumulation measurements by considering the impact of the synthetic exposure medium (PPYE) on the accumulation. These controls revealed that accumulation measurements were reliable: free‐copper controls (0 mg/L) showed that there was no detectable copper in the PPYE and the controls with copper (150, 275 and 350 mg/L) showed that the concentration of copper placed in the medium was entirely found in the medium after 96 h under all thermal conditions (Table S1). As a result, the measurements of copper found in the medium after filtration of the cells correspond to the copper that was not accumulated by the cells. This study measures accumulation as a whole throughout the manuscript, thus considering both internal and external accumulation simultaneously, because our objective was to analyse the effects of global accumulation rather than focusing on the effects linked to the type of accumulation.

### Statistical analysis

We performed all statistical analyses on R version 4.2.2 (R Core Team, 2022).

First, the effects of copper concentration gradient on demography, morphology, swimming and accumulation traits at the different thermal conditions were determined by generalised linear models (GLMs) using the R‐stats package: *glm* function: *trait* ~ *concentration* + *temperature* + *strain* + *concentration* × *temperature + concentration* × *strain* + *temperature* × *strain* + *concentration* × *temperature* × *strain*. The normality of each trait distribution was verified using Shapiro–Wilk normality test. Copper concentration was considered as a discrete variable, and temperature and strain were considered as categorial variables. Indeed, this allows us to detect significant differences between each of the three temperatures, given that we had no specific argument justifying linear or quadratic effects of temperature. For accumulation estimation, copper treatments at 0 mg/L were excluded since there cannot be copper accumulation in a copper‐free medium. All numeric response variables have been centred and scaled to obtain a mean of 0 and a standard deviation of 1 to allow comparisons of effects between all traits and strains. The final models were selected according to a backward selection method on the basis of the Akaike information criterion. In cases of significant effects involving at least one categorical variable, contrast tests were carried out in order to determine the intensity and direction of the observed effects. We applied a false discovery rate pairwise comparison correction to determine the significance level of the effects.

Secondly, we aimed at studying if accumulation may influence the effects that copper and temperature have on the other phenotypic traits. To determine how to implement this analysis, that is, a global model integrating all strains and temperatures or by separating each strain and/or temperature, we first analysed if strain identity and temperature had an influence on the relationships between phenotypic traits and accumulation. To do so, we calculated the effect size of the correlation between accumulation concentration (as we wanted to focus on the amount of copper accumulated by strains) and each of the other trait (demography, size, elongation and velocity). Effect sizes allow us to quantify the magnitude of a difference or a relationship between many variables. We used the *datawizard* package (Patil et al., 2022) and the *effectsize* package (Ben‐Shachar et al., 2020) to run linear models (LM) in the form of *estimate* (*trait* ~ *accumulation*) ~ *strain* + *temperature* + *strain* × *temperature using standardised parameters*. *Estimate(trait ~ accumulation)* was the estimate value resulting from LMs in the form of *trait ~ accumulation* considering the three replicates of trait values for each of the six strains and each of the three temperatures separately. We thus obtained 18 values of effect size per trait. Significant variables were determined through a backward selection procedure. Backward selection is one of the main approaches to stepwise regression (Hocking, 1976). It consists of the deletion of variables whose loss does not result in a statistical deterioration of the fit of the model. The variable removal process is repeated until no more non‐significant variables in the model fit. This effect size analysis revealed a significant effect of strain × temperature on at least one trait (see Results section). This means that the influence of accumulation on the response of the other traits to copper and temperature has to be studied for each strain and temperature separately.

The links between accumulation concentration and the other phenotypic traits were studied using structural equation modelling analysis (SEM) with the *Lavaan* package (Rosseel, 2012). SEM analysis consists of the creation of a single model that represents all the relationships (expressed in the form of equations) between the measured variables. It combines regression relations and path analyses to estimate relationships between chosen predictor variables and response variables (Grace et al., 2015; Kline, 2016; Schreiber et al., 2006). SEM involves estimating the coefficients of equations to determine the intensity and direction of links between different variables. Thus, it allows us to distinguish the parts of the phenotypic variance attributed to the copper concentration gradient from those attributed to the copper accumulation of the strains. In the end, it provides prediction on the participation of accumulation in the tolerance of strains to copper at different temperatures. Our models combined copper concentration gradient and copper accumulation, called ‘concentration’ and ‘accumulation,’ respectively, as predictors; and demography (‘Δ_cells_’), cell size (‘size’), cell elongation (‘elongation’) and swimming speed (‘velocity’) as response variables to be predicted by the same pathway to test different scenarios involving regression relationships (Bollen, 1989; Jorgensen & Jak, 2020; Rosseel, 2012). The factor variable ‘strain’ was considered a group and the analysis was carried out separately for each of the exposure temperatures (see above). Models' accuracies were evaluated using several criteria: *χ* ^2^, the root mean square error of approximation, the standardised root mean square residual and the comparative adjustment index (Fan et al., 2016). For these models, *p* > 0.05 reflects good quality of fit. Criterion to assess the accuracy of these SEM models showed satisfactory quality of fit (Table S3).

## RESULTS

### Effects of copper and temperature on demography and phenotypic traits

#### Demography

**FIGURE 1:**
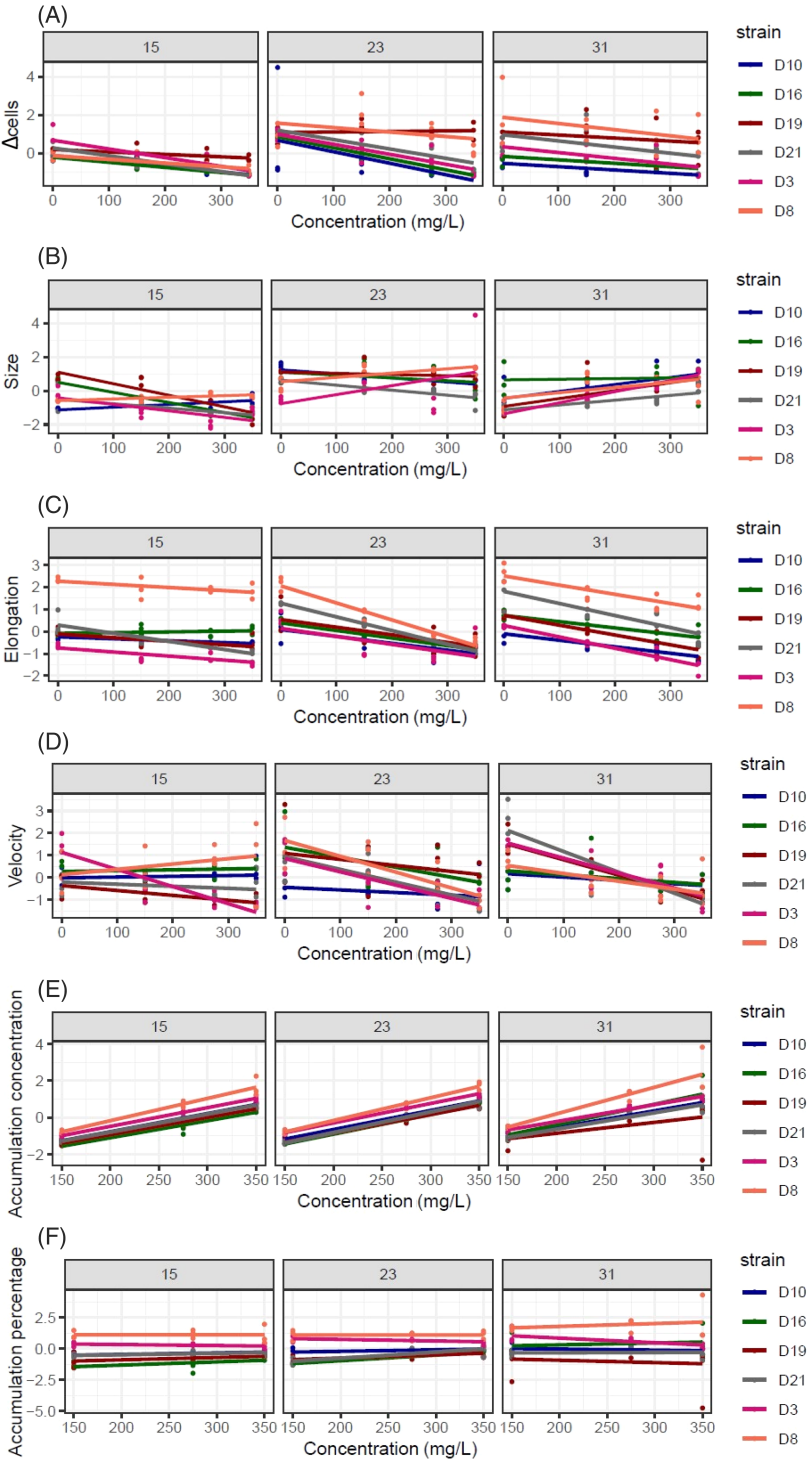
Copper effects on phenotypic traits (A) demography, (B) size, (C) elongation, (D) velocity, (E) accumulation concentration and (F) accumulation percentage along the thermal gradient. Colours represent each of the six strains. Points represent the values (three replicates per strain) of each trait at the corresponding copper concentration. The lines are the regressions obtained from the glm models. Error margins are not displayed for better readability of the graphs (observation of the margins of error are available in Figure S5).

For all strains, copper concentration had a negative effect on Δ_cells_ (Figure 1A; Table 1, see all curves with their standard deviation in Figure S5). The interaction strain × temperature was significant (Table 1). For all strains, Δ_cells_ was the lowest at the cold temperature (15°C) and at the warmer temperature (23 and 31°C), and D8 and D19 had higher Δ_cells_ than the four other strains (Table S2). Noteworthy, temperature did not affect the copper impact on population Δ_cells_, whatever the strain (non‐significant copper × temperature and copper × temperature × strain interactions, Table 1), although we observed a tendency for a lesser variance in the responses to copper stressor at 15°C between strains.

**TABLE 1:** Results presenting the effect significance of copper concentration on demography, size, elongation, and swimming velocity for each strain under the three exposure temperatures 15, 23 and 31°C. Generalised linear models (analysis of variance): *trait* ~ *concentration* + *temperature* + *strain* + *concentration* × *temperature* + *concentration* × *strain* + *temperature* × *strain* + *copper concentration* × *temperature* × *strain*. Significant terms are highlighted in bold.

Trait	Deviance *p*‐value						
	Copper concentration	Temperature	Strain	Copper concentration × strain	Copper concentration × temperature	Strain × temperature	Copper concentration × temperature × strain
Δ_cells_	**37.62**	**21.4**	**54.7**	5.4	1.5	**18.5**	3.6
*n* = 212	**0.04**	**<0.0001**	**<0.0001**	0.1	0.15	**<0.0001**	0.53
Size	**0.6**	**65.93**	**21.36**	**11.93**	**24.28**	**7.41**	**15.29**
*n* = 212	**0.001**	**<0.039**	**<0.0001**	**<0.0001**	**<0.0001**	**0.02**	**<0.0001**
Elongation	**41.92**	**4.5**	**106.23**	**4.57**	**6.95**	**21.68**	2.18
*n* = 212	**<0.0001**	**<0.0001**	**<0.0001**	**<0.0001**	**<0.0001**	**<0.0001**	0.09
Velocity	**32.87**	**7.29**	**27.15**	**15.20**	6.47	**27.28**	**13.82**
*n* = 212	**<0.0001**	**<0.0001**	**<0.0001**	**<0.0001**	0.9	**<0.0001**	**<0.0001**
Accumulation concentration	**112.8**	2	**0.235**	**21.4**	2.13	3.19	0.948
*n* = 161	**<0.0001**	0.25	**<0.0001**	**0.01**	0.41	0.18	0.71
Accumulation percentage	0.75	6.21	**89.64**	1.10	2.85	11.81	1.44
*n* = 161	0.15	0.09	**<0.0001**	0.17	0.22	0.08	0.95

#### Morphology and swimming traits

Copper had significant effects on cell size, cell elongation and swimming velocity, although the direction of these effects changed between strains and was temperature‐dependent (significant copper × temperature × strain interaction, except for elongation, for which only the two‐way interactions were significant Table 1). Cell size generally decreased along increasing copper concentration at 15°C, while this pattern reversed at 31°C. However, this pattern differed in some strains (see, for instance, D8 at 15°C and D16 at 31°C, Figure 1B; Tables 1 and S2). At 23°C, a high variation in the effects of copper on cell size was observed between strains. Cell elongation also changed depending on copper, temperature and strain, with cells being generally rounder as copper concentration increased (Figure 1C), an effect that was stronger in some strains and some temperatures (for instance, D8 at 23°C) and non‐significant at 15°C for half of the strains. Finally, velocity decreased as copper concentration increased for all strains at 23 and 31°C (Figure 1D). At 15°C, cell velocity either increased, decreased or did not vary as copper concentration increased, depending on the strain (Figure 1D).

#### Accumulation traits

Contrary to the other traits, accumulation traits (accumulation amount and accumulation percentage) did not depend on temperature (Table 1). Accumulation concentration increased with the concentration of copper in the media for all strains (Figure 1E; significant copper concentration effect; Table 1), D3 and D8 accumulating the most (significant strain effect; Tables 1 and S2), especially as copper concentration increased (significant concentration × strain interaction; Tables 1 and S2). Accumulation percentage remained constant regardless of the copper concentration in the media (Figure 1F; 25% ± 5% over all strains) and was higher for D3 and D8 (significant strain effect, Tables 1 and S2). Surprisingly, among the two strains that tolerated better copper over all temperatures (i.e., higher Δ_cells_ for D8 and D19), only D8 had among the highest accumulation traits, while D19 had among the lowest.

### Links between accumulation and the other phenotypic traits

We first studied the impact of strain identity and temperature on the effect sizes obtained from the models, testing for a correlation between accumulation concentration and the other traits (Figure S6). We detected a significant effect of the interaction between temperature and strain on demography (*p* < 0.0001), a significant effect of strain on elongation (*p* < 0.0001), and a marginal effect of temperature on size (*p* = 0.039; Table 1). As a result, SEM analyses, which aimed at studying the links between accumulation concentration and all the other phenotypic traits in a unique path model, were conducted for each temperature and strain separately.

#### Correlation between accumulation and demography

**FIGURE 2:**
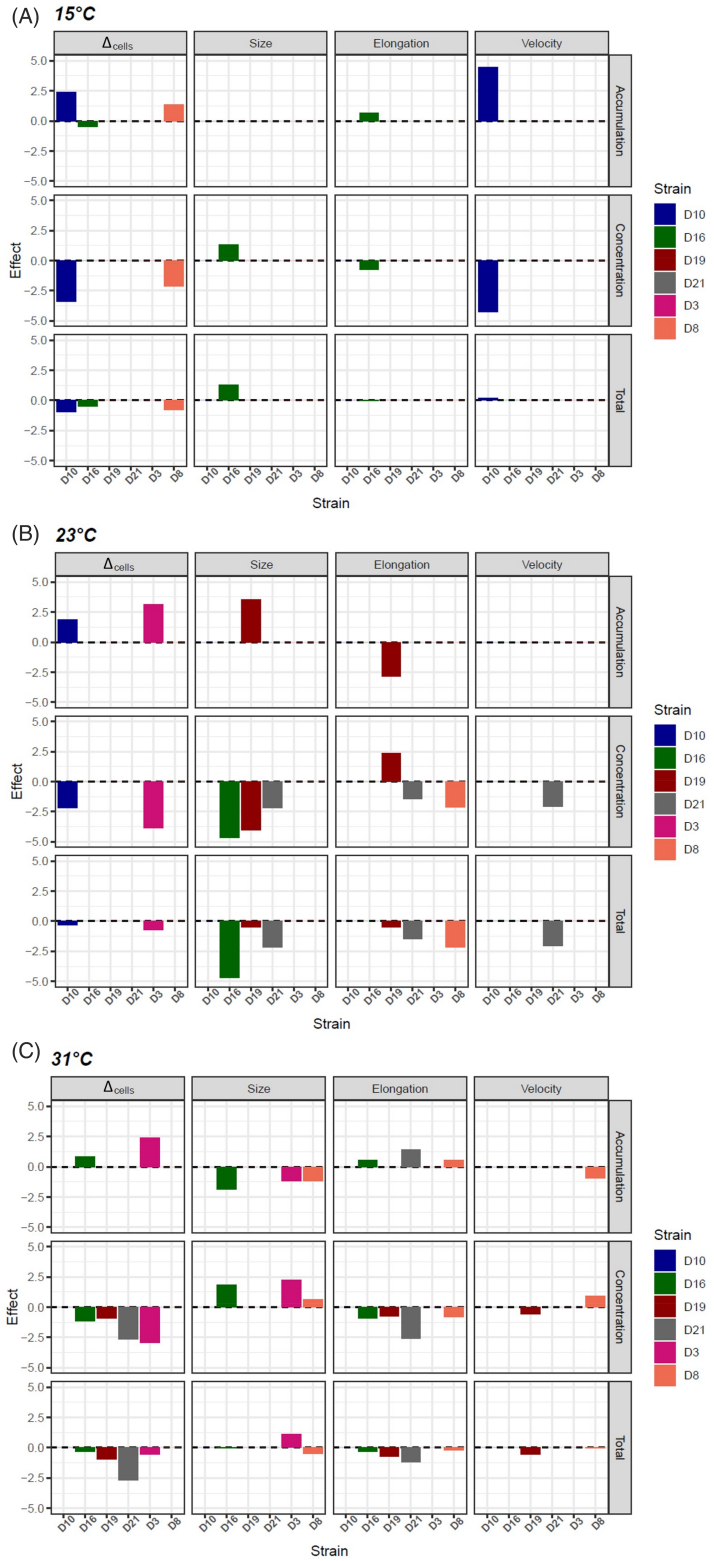
Copper concentration, accumulation level and total effects of copper on demography, cell size, cell elongation and swimming velocity for each strain (colour bars) at each temperature (A) 15°C, (B) 23°C and (C) 31°C as obtained from SEM. Concentration effects are estimated from the relationship between copper concentration and phenotypic traits. Accumulation effects are estimated from the sums of the products of the path coefficients from copper to accumulation, then from accumulation to the phenotypic trait. Total effects correspond to the sum of concentration and accumulation effects. Only significant effects are represented by the colour bars (*p* < 0.05).

The correlation between copper concentration and Δ_cells_ was negative (Figure 2; Table S4 for individual SEM results, see Figure S7 for a representation of all the pathways of effects for all traits, temperatures and strains), as previously demonstrated by the GLM analyses, which indicates deleterious effects of copper on demography. The correlation between accumulation and Δ_cells_ was positive in some strains at specific temperatures, suggesting that accumulation significantly attenuated the inhibitory effects of copper on demography. Indeed, accumulation significantly attenuated Δ_cells_ decrease for D8 at 15°C, D10 at 15°C and 23°C, D3 at 23°C and 31°C and D16 at 31°C (Figure 2). The only significant negative correlation between accumulation and Δ_cells_ was for D16 at 15°C. Of note, in D19, there was no significant correlation between copper accumulation and Δ_cells_ (Figure 2), while this strain had the highest Δ_cells_ over all copper concentrations and temperatures.

#### Contrasted effect of copper concentration and accumulation

In ten cases, the correlations between copper accumulation and the other traits on the one hand, and between copper concentration and these traits on the other hand, were significant and opposite (Figure 2). When copper concentration tended to increase cell size (e.g., in D16 at 23 and 31°C) and reduce cell elongation (e.g., D21 at 23 and 31°C) and cell velocity (e.g., D10 at 15°C), accumulation tended to attenuate these effects, leading to smaller total copper effects (Figure 2). However, in nine other cases, there were only copper concentration effects without the involvement of accumulation. For example, at 23°C, accumulation did not correlate with the reduction in cell size of D16 and D21 or the fact that the cells became rounder for D8 and D21 (Figure 2).

## DISCUSSION

In this study, we tested the combined effects of copper and temperature on the demography, morphological, swimming and accumulation traits of *T. thermophila*, and estimated intraspecific variability in these phenotypic responses. Analyses showed that copper modified the demography of strains, their morphology, as well as their swimming behaviour, depending on thermal conditions. The level of copper accumulation increased along with the copper concentration gradient, independent of temperature. Results also indicated that there was intraspecific variability in all measured phenotypic traits (significant strain effect). It is noteworthy that these differences between strains were modulated by temperature and/or copper concentration. A notable exception was the accumulation percentage, which was different between strains but unaffected by either of our experimental treatments. To better understand how accumulation is involved in the observed responses to the combination of copper and thermal stressor, we ran path analysis linking accumulation to all other traits (Δ_cells_, size, elongation and velocity). While we revealed attenuating effects of accumulation on copper effects in some strains at specific temperatures, it was not a general pattern. We observed that accumulation had no significant effect on the demography of the most copper‐tolerant strain (D19), whatever the temperature. We shall discuss these findings in light of the cellular mechanisms that could explain the revealed patterns.

### Temperature‐dependent effects of copper concentration on cellular characteristics

In agreement with previous studies on *Tetrahymena* species, which showed that copper can have lethal effects and modify the morphology and swimming behaviour of cells (e.g., Arcila et al., 2019; Dayeh et al., 2005; Kumar et al., 2014; Nicolau et al., 1999; Nilsson, 1981), we found that copper had significant effects on *T. thermophila* demography and phenotypic traits. We observed that the changes in the number of cells over 96 h, our proxy of fitness, were dependent on copper and temperature, but we did not detect any significant interaction between the two stressors: Δ_cells_ decreased along with increasing copper and decreasing temperature. Moreover, we found that temperature could modulate copper effects on cell elongation, size and swimming velocity. Indeed, cells were generally smaller and rounder as copper concentration increased in the lower part of the thermal gradient, but at the warmer temperature, cells generally became larger (notice that some strains deviate from these general patterns). The thermal‐dependency was different for swimming velocity, which decreased for the majority of strains at the two warmer temperatures as copper concentration increased, while it could go in all directions at the lower temperature. Such changes in demography, cell morphology and swimming might be due, at least in part, to membrane alteration and inhibition of proteins in the presence of copper (Somasundaram et al., 2018) and at non‐optimal temperature (DeLong et al., 2017; Weber de Melo et al., 2020). In general, the strains of *T. thermophila* studied have thermal optima between 26 and 32°C (Jacob et al., 2018; Figure S2). Our coldest temperature (15°C) is thus far from these optimal conditions. Cold generates a heterogeneous and disturbed distribution of *Tetrahymena* membrane particles, thus inhibiting membrane fluidity and altering lipid composition (Nozawa, 2011). Indeed, the membrane phospholipids move less and become more rigid. This decreases the overall fluidity of the membrane, also decreasing its permeability and potentially restricting the entry of important molecules such as glucose into the cell. For instance, it is known that cold thermal stress can alter the membrane of *T. thermophila* by degrading the integral and peripheral proteins in the membrane responsible for cell morphology (Sanchez Granel et al., 2019). Cold temperature can also decrease cell survival and growth by reducing cell size (Chandler, 2023). This would explain the strong effects of cold temperature (15°C) on the demography of strains and morphology, and perhaps the tendency towards more homogenous responses at cold temperature (thermal stressor so high that copper effects are less effective). However, how cells' demography, morphology and swimming are affected by the interaction between copper and temperature is unknown in *T. thermophila*. As strains differed in their response to the interaction between copper and temperature, mechanistic investigations on multiple strains will be required to determine what drives the morphological and swimming effects observed in this study (Table 2).

**TABLE 2:** Results of the backward selection from models linking effect sizes of trait ~ accumulation relationship to genotype and temperature (*effect size trait* ~ *strain* + *temperature* + *strain* × *temperature*). Only significant variables (in bold) after backward selection are presented. Velocity is not included as it was not affected by strain nor temperature. Temperature has a marginal effect on the effect size of cell size.

Variables	Effect size_Δ_cells_		
	*F*‐value	Df	*p*‐Value
Strain	6.9684	5	**<0.0001**
Temperature	0.0737	1	0.787397
Temperature × strain	4.1264	5	**0.003873**

Variables	Effect size_size		
	*F*‐value	Df	*p*‐Value
Temperature	3.335	1	0.0866

Variables	Effect size_elongation		
	*F*‐value	Df	*p*‐Value
Strain	3.647	5	**0.0308**

Surprisingly, temperature had no significant effect on accumulation, neither through simple nor through interactive effects. This result contrasts with other studies looking at accumulation of copper, zinc and cadmium by ciliates (Liaqat et al., 2022; Martín‐González et al., 2006). One explanation could be that our strains favour an accumulation strategy that is not very sensitive to temperature. Morillo‐Pérez et al. (2008) and Velásquez and Dussan (2009) showed that many microorganisms rely mainly on external accumulation, which requires a very low energy supply. This passive accumulation pathway consists in the formation of metal ion complexes on the cell membrane by the receptors. As temperature has no direct effect on the polarity of membrane surface receptors below 50°C (Geddie & Sutherland, 2008), it could explain why temperature had no significant effect on accumulation level in our study. In the future, analyses of transcriptomes after exposure to temperature and copper stressors would help to characterise the genes involved in the accumulation ability of different strains of *T. thermophila*. This could also help shed light on the mechanisms responsible for the intriguing result of an equal percentage of accumulation observed, whatever the copper concentration in the medium.

### Accumulation can mitigate the toxic effects of copper, but other mechanisms are also at play

Accumulation ability is a well‐known resistance mechanism in ciliates (and other taxa) to better tolerate and detoxify certain heavy metals (Martín‐González et al., 2006; Peng et al., 2019). It can participate in reducing the toxic effects of copper, so it might mediate part of the observed effects of copper. Our SEM analysis suggested that accumulation may reduce the deleterious effects of copper on demography in some strains because, when significant, the direction of its effects was opposite to those of the effects of copper concentration. This effect seems general in ciliates. Indeed, accumulation helps a strain of *Paramecium multimicronucleatum* to resist copper (Liaqat et al., 2022). This strain was able to accumulate more than 80% of copper in the medium, and this copper accumulation induced an increase in the production of glutathione and thiols, which in turn contributed to the reduction of oxidative stress caused by copper (Liaqat et al., 2022). In the genus *Tetrahymena*, the strains that accumulate the most are usually those that resist copper the best (Chaudhry & Shakoori, 2011). However, in our results, some strains showed no involvement of accumulation in their demography but had nonetheless good copper tolerance. This was the case of D19, which had the highest number of cells after 96 h over all copper concentrations and temperatures, despite no significant correlation between its demography and accumulation. This strain could have other resistance mechanisms, for instance avoidance strategies such as cell efflux, which consists of expelling excess copper from the cell after entry (Solioz & Stoyanov, 2003). This mechanism would also help maintain cell homeostasis when they are exposed to heavy metals. Cell efflux is often facilitated by efflux transport proteins that act as pumps to remove metals out of the cell (Nies, 2003; Nies & Silver, 1995). Strains expressing this strategy would putatively remove very little copper in the exposure medium (~0.5% of the copper present in the medium in some species; Kaduková & Virčíková, 2005) while resisting well to copper. Our experiments cannot make conclusions on the nature of the other mechanisms involved in copper tolerance. However, they clearly show that several tolerance mechanisms can be at play within the same species, but with strong differences between strains.

### Importance of intraspecific variability in ecotoxicological studies

In this study, although we have evaluated the responses of only six strains of *T. thermophila* exposed only to two stressors (copper and temperature) and analysed only five phenotypic traits (cell size and cell elongation, swimming velocity, copper accumulation percentage and copper accumulation level) and one proxy of fitness, our observations have revealed substantial intraspecific variation. Demography differed considerably between strains, morphological and swimming traits went in all directions depending on temperature, and copper accumulation varied between strains but was not affected by temperature. This complexity suggests that biological functions react in a very specific way to stress conditions, depending not only on the genetic combination of each strain, but also on the interaction between stressors. For example, the study of Delnat et al. (2022) on six strains of *Daphnia magna* exposed to the combined effect of temperature and pesticide revealed variations in the interactive effects of the two stressors according to the strain and the trait considered. Regarding growth, four *Daphnia* strains showed antagonistic effects, one strain showed additive effects and one presented synergistic effects. In terms of maturation time, two strains displayed synergistic effects, two others showed additive effects, while the last two presented antagonistic effects (Delnat et al., 2022).

Our results showed that accumulation effects on phenotypic trait changes were not always significant, depending on strain × temperature combinations. Our analysis also highlighted that the strains presenting the highest accumulation were not systematically the most tolerant to the toxic effects of copper. For example, D3 and D8 were the most copper‐accumulative, while D19 was among the least accumulative. However, D8 and D19 resisted the best to copper. Therefore, copper accumulation level might not be the only resistance mechanism to copper expressed by our strains. The tolerance of strains probably results from various genomic mechanisms (allelic variation and gene expression) interacting in complex ways to produce unique expression profiles reflecting their degree of tolerance. A study in 1500 strains of *Drosophila melanogaster* showed that resistance to the toxic effects of copper is genetically complex and influenced by the allelic and expression variation in several loci, which could produce strain‐specific expression profiles and thereby variability of tolerance (Everman et al., 2021).

As in our study, recent years have revealed that the results of ecotoxicological studies, especially on freshwater ecosystems, can strongly differ between populations (Delnat et al., 2022; Petitjean et al., 2021; Roubeau Dumont et al., 2019). In some cases, intraspecific variation may even have equivalent or greater influence than that of interspecific differences on overall community responses to anthropogenic stressors (Jacob & Legrand, 2021; Raffard et al., 2019). However, it is noteworthy that intraspecific variability is only poorly considered in environmental risk analyses, while it is an important component of (freshwater) ecosystems and is a crucial factor in implementing adequate management strategies (Brady et al., 2017). This study supports this claim, as we highlighted that the sensitivity and tolerance mechanisms to copper and temperature can be highly different between different *T. thermophila* strains.

## AUTHOR CONTRIBUTIONS

**Doufoungognon Carine Estelle Koné:** Conceptualization (lead); data curation (lead); formal analysis (lead); methodology (equal); software (equal); writing – original draft (lead); writing – review and editing (lead). **Staffan Jacob:** Formal analysis (supporting); methodology (supporting); software (supporting); writing – review and editing (supporting). **Michèle Huet:** Methodology (lead); supervision (supporting). **Hervé Philippe:** Conceptualization (supporting); methodology (supporting); software (supporting); supervision (supporting); writing – review and editing (supporting). **Delphine Legrand:** Conceptualization (lead); funding acquisition (lead); methodology (equal); project administration (lead); resources (equal); software (supporting); supervision (lead); validation (lead); writing – original draft (supporting); writing – review and editing (supporting).

## CONFLICT OF INTEREST STATEMENT

The authors declare that they have no known competing financial interests or personal relationships that could have appeared to influence the work reported in this paper.

## Supporting information


**Data S1.** Supporting Information.

## Data Availability

The data supporting the findings of this study is available from the Dryad Digital Repository: https://doi.org/10.5061/dryad.f7m0cfz36.
