# Supplementary material for: The phenotypic and demographic response to the combination of copper and thermal stressors strongly varies within the ciliate species, Tetrahymena thermophila
Source: Environ Microbiol Rep. 2024 Sep 30;16(5):e13307. doi: 10.1111/1758-2229.13307 (PMC11440147; doi:10.1111/1758-2229.13307)
Supplement: Supplementary file 1 — Data S1. Supporting Information. [file EMI4-16-e13307-s001.docx]

**Supplementary Material:** The phenotypic and demographic response to the combination of copper and thermal stressors strongly varies within the ciliate species, *Tetrahymena. thermophila*

Doufoungognon Carine Estelle Koné^1,*^, Michèle Huet^1^, Staffan Jacob^1^, Hervé Philippe^1^, Delphine Legrand^1,*^

^1^Station d'Ecologie Théorique et Expérimentale, UAR2029, 2, route du CNRS, 09200, Moulis, France.

* Corresponding authors

*Figure S1: Pilot experiment aimed at determining the copper tolerance of each* Tetrahymena thermophila *strain under our laboratory conditions (23°C).*


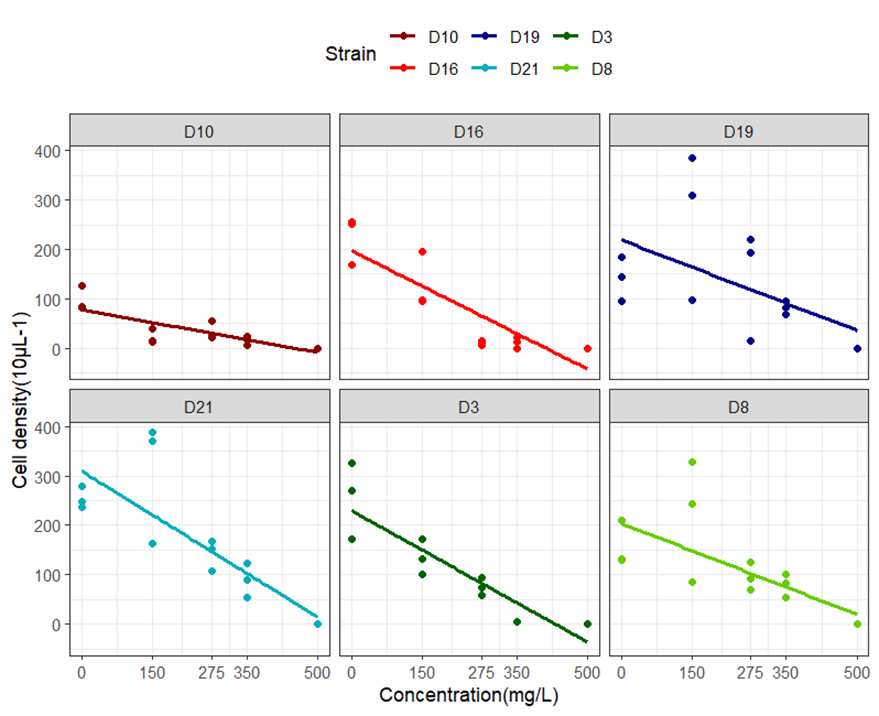


For three replicates of each strain and each tested copper concentration (0, 150, 275, 350 and 500 mg/L), 2.5mL of 7-day axenic culture (~ 59,783 ± 16,450 cells overall strains) were inoculated into 50mL tubes previously filled with 10mL of the adequate exposure medium. After 96h of culture (4 days), we estimated the number of cells for each tested condition. Without copper, 235,694 ± 75,066 cells were counted overall strains, while at 500mg/L, no cells were detected in either of the six tested strains. As living cells were observed at 350mg/L (58,680 ± 45,532 cells overall strains), we fixed this latter concentration as the maximal tolerable concentration in our experiment.

*
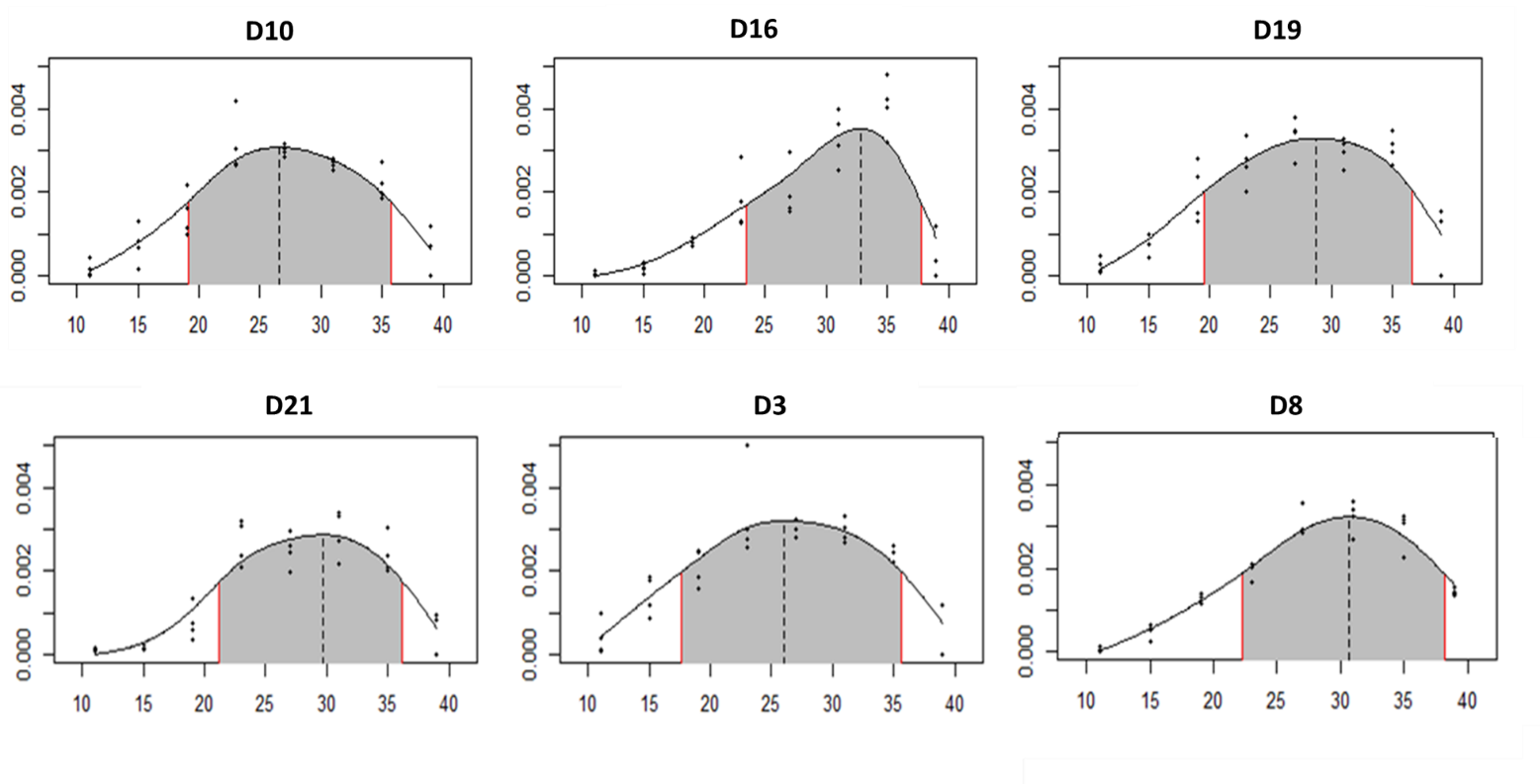
Figure S2: Thermal tolerance curve under our standard conditions (PPYE medium without copper)*

For each strain, the growth rate at eight temperatures (11, 15, 19, 23, 27, 31, 35 and 39°C) were estimated by inoculating 10µL (~100 cells) in 240µL of PPYE medium within 96-well plates under four replicates. Population growth was measured through absorbance at 450 nm twice a day during 15 days using a microplate reader (Tecan Infinite Spectrophotometer). Spline-based growth curves were fitted using the grofit R-package (*gcfit* function; Kahm et al., 2010) and growth rates were computed as the maximum slopes of population growth as in previous studies of our group (Jacob et al., 2017, 2018; Jacob & Legrand, 2021). The black lines on the graphs correspond to the fit of a general additive model of growth rate as a function of temperature. The grey areas represent niche width (the temperature range containing 80% of the thermal niche), and the dashed line shows estimated thermal optimum.

*
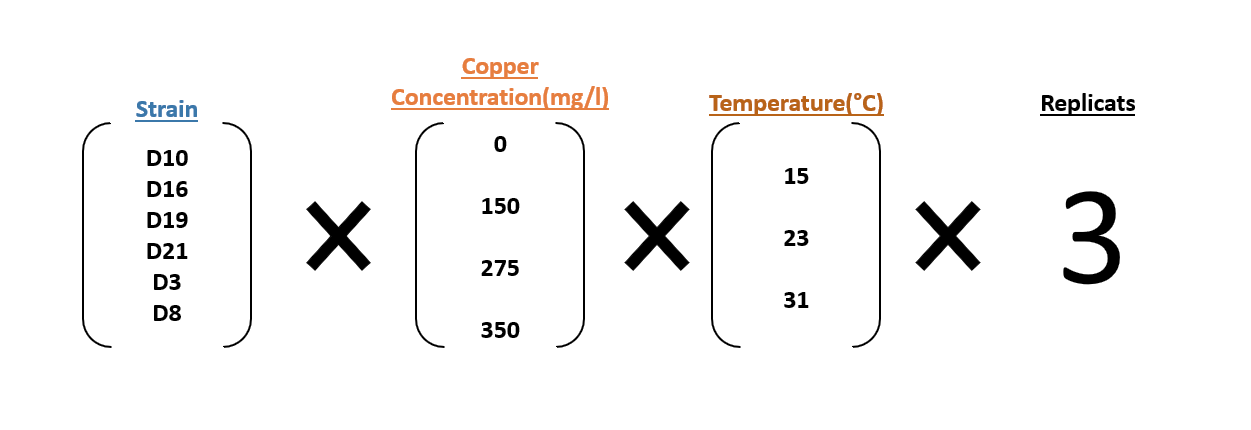
Figure S3: Experimental design of the whole experiment*

*Figure S4: Experimental design for the estimation of copper concentration by ICP-AES in free of cell controls*


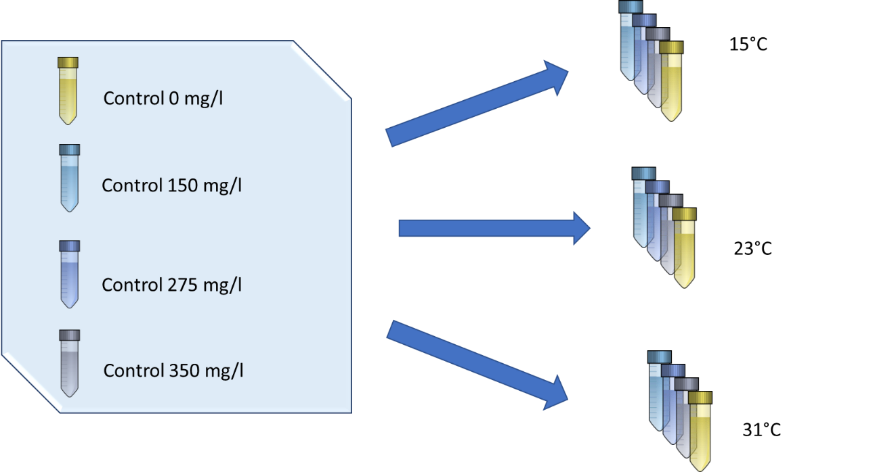


*Table S1: Table of standard error of measurement of controls by ICP-AES after 96h*

| Temperature (°C) | Copper concentration supposed to be obtained after medium preparation | Standard Errors estimated by ICP-AES |
| --- | --- | --- |
| 31 | 150 | 0.0018 |
| 31 | 275 | 0.0048 |
| 31 | 350 | 0.0042 |
| 31 | 0 | 0.00006 |
| 23 | 0 | 0.0002 |
| 23 | 350 | 0.0041 |
| 23 | 275 | 0.0016 |
| 23 | 150 | 0.0012 |
| 15 | 0 | 0.00006 |
| 15 | 350 | 0.0019 |
| 15 | 275 | 0.0017 |
| 15 | 150 | 0.0023 |

*Table S2: Results of the contrast analyses of the GLMs (ANOVA) for each phenotypic trait. Dashes represent non-significant effects (p>0.05). Compared references: strain D10; temperature: 15°C*

| ***Variables*** | ***Δcells*** | ***Size*** | ***Elongation*** | ***Velocity*** | ***Accumulation concentration*** | ***Accumulation percentage*** |
| --- | --- | --- | --- | --- | --- | --- |
|  | ***Estimate ± SE***  ***P*** | ***Estimate ± SE***  ***p*** | ***Estimate ± SE***  ***p*** | ***Estimate ± SE***  ***p*** | ***Estimate ± SE***  ***p*** | ***Estimate ± SE***  ***p*** |
| **Concentration** | -0.38 ± 0.18  **0.04** | -0.21 ± 0.17  **<0.0001** | -0.11 ± 0.10  **<0.0001** | -0.05 ± 0.19  **<0.0001** | 1.30 ± 0.11  **<0.0001** | _  _ |
| **Temperature** |  |  |  |  |  |  |
| Temperature 23 | 1.04  **<0.0001** | 1.59 ± 0.24  **0.001** | --  -- | -0.64 ± 0.27  **0.02** | - | _  _ |
| Temperature 31 | 1.67  **<0.0001** | 1.17±0.24  **0.001** | -0.26 ± 0.14  **0.08** | -  - | _ | _  _ |
| **Strain** |  |  |  |  |  |  |
| StrainD16 | _  _ | -  - | -0.26 ± 0.15  **0.01** | -  - | -  - | -  - |
| StrainD19 | 0.62 ± 0.25  **0.017** | 0.59 ± 0.24  **0.01** | _  _ | -0.75 ± 0.27  **0.006** | _  _ | _  _ |
| StrainD21 | _  _ | _  _ | _  _ | _  _ | _  _ | _  _ |
| StrainD3 | _  _ | _  _ | -0.68 ± 0.15  **0.0001** | _  _ | 0.56 ± 0.28 **0.006** | 0.68 ± 0.28  **0.018** |
| StrainD8 | 0.47 ± 0.25  **0.07** | 0.43 ± 0.24  **0.08** | 2.4 ± 0.15  **0.0001** | -0.48±0.27  **0.08** | 0.52 ± 0.18  **0.005** | 1.52 ± 0.28  **<0.0001** |
| **Concentration : Temperature** |  |  |  |  |  |  |
| Concentration : Temperature 23 | _  _ | 0.53 ± 0.24  **0.03** | -0.29 ± 0.15  **0.04** | --  -- | _  _ | _  _ |
| Concentration : Temperature 31 | _  _ | --  -- | -0.27 ± 0.15  **0.06** | --  -- | _  _ | _  _ |
| **Concentration : Strain** |  |  |  |  |  |  |
| Concentration : StrainD16 | _  _ | -1 ± 0.24  **0.0001** | _  _ | _  _ | _  _ | _  _ |
| Concentration : StrainD19 | _  _ | -1.12 ± 0.24  **<0.0001** | _  _ | _  _ | _  _ | _  _ |
| Concentration : StrainD21 | _  _ | -0.56 ± 0.24  **0.02** | -0.37 ± 0.15  **0.01** | _  _ | _  _ | _  _ |
| Concentration : StrainD3 | _  _ | -0.72 ± 0.24  **0.004** | _  _ | 0.96 ± 0.27  **0.006** | 0.35 ± 0.18  **0.03** | _  _ |
| Concentration : StrainD8 | _  _ | _  _ | _  _ | _  _ | 0.42 ± 0.16  **0.008** | _  _ |
| **Temperature : Strain** |  |  |  |  |  |  |
| Temperature 23 : StrainD16 | _  _ | _  _ | _  _ | 0.77 ± 0.39  **0.04** | _  _ | _  _ |
| Temperature 31 : StrainD16 | _  _ | _  _ | 0.45 ± 0.21  **0.03** | 1.6 ± 0.39  **0.0001** | _  _ | _  _ |
| Temperature 23 : StrainD19 | 1 ± 0.36  **0.007** | _  _ | 0.40 ± 0.21  **0.05** | 1.86 ± 0.39  **0.0001** | _  _ | _  _ |
| Temperature 31 : StrainD19 | 1.05 ± 0.36  **0.004** | -0.97 ± 0.35  **0.006** | 0.55 ± 0.21  **0.009** | 0.99 ± 0.39  **0.01** | _  _ | _  _ |
| Temperature 23 : StrainD21 | _  _ | _  _ | 0.60 ± 0.21  **0.004** | 0.84 ± 0.39  **0.03** | _  _ | _  _ |
| Temperature 31 : StrainD21 | _  _ | -0.73 ± 0.35  **0.03** | 1.43 ± 0.21  **0.0001** | 0.76 ± 0.39  **0.05** | _  _ | _  _ |
| Temperature 23 : StrainD3 | _  _ | _  _ | 0.63 ± 0.21  **0.003** | 0.69 ± 0.39  **0.08** | _  _ | _  _ |
| Temperature 31 : StrainD3 | _  _ | _  _ | 0.63 ± 0.21  **0.004** | 0.69 ± 0.39  **0.08** | _  _ | _  _ |
| Temperature 23 : StrainD8 | 1.43 ± 0.36  **0.0001** | _  _ | -1.31 ± 0.21  **0.0001** | _  _ | _  _ | _  _ |
| Temperature 31 : StrainD8 | 1.95 ± 0.36  **0.0001** | -0.60 ± 0.35  **0.08** | _  _ | _  _ | _  _ | _  _ |
| **Concentration : Temperature : Strain** |  |  |  |  |  |  |
| Concentration : Temperature 23 : StrainD16 | _  _ | 1.10 ± 0.35  **0.002** | _  _ | _  _ | _  _ | _  _ |
| Concentration : Temperature 31 : StrainD16 | _  _ | --  -- | _  _ | -0.40 ± 0.39  **0.009** | _  _ | _  _ |
| Concentration : Temperature 23 : StrainD19 | _  _ | 1.34 ± 0.35  **0.001** | _  _ | _  _ | _  _ | _  _ |
| Concentration : Temperature 31 : StrainD19 | _  _ | 1.19 ± 0.35  **0.0001** | _  _ | _  _ | _  _ | _  _ |
| Concentration : Temperature 23 : StrainD21 | _  _ | _  _ | _  _ | _  _ | _  _ | _  _ |
| Concentration : Temperature 31 : StrainD21 | _  _ | _  _ | _  _ | -0.79 ± 0.39  **0.04** | _  _ | _  _ |
| Concentration : Temperature 23 : StrainD3 | _  _ | 1.75 ± 0.35  **0.0001** | _  _ | _  _ | _  _ | _  _ |
| Concentration : Temperature 31 : StrainD3 | _  _ | 1.04 ± 0.37  **0.005** | _  _ | _  _ | _  _ | _  _ |
| Concentration : Temperature 23 : StrainD8 | _  _ | 0.74 ± 0.35  **0.03** | _  _ | -0.95 ± 0.39  **0.01** | _  _ | _  _ |
| Concentration : Temperature 31 : StrainD8 | _  _ | _  _ | _  _ | _  _ | _  _ | _  _ |

*Table S5: Comparison table of P values from glm models and the p values adjusted by the FDR pairwise comparison method*

| **Trait** | **Variables** | **P value GLM** | **P adjust FDR method** |
| --- | --- | --- | --- |
| **Δcells** | (Intercept) | 0.0004685363143123 | 0.002225547492983 |
|  | concentration | **0.042428988275928** | **0.05387018126624** |
|  | temperature23 | 0.54387018126624 | 0.653253595657988 |
|  | temperature31 | 0.42902552797316 | 0.653253595657988 |
|  | strainD16 | 0.846114055413874 | 0.893120391825756 |
|  | strainD19 | **0.017441733215680** | **0.048549116387240** |
|  | strainD21 | 0.566594847793623 | 0.672831381754927 |
|  | strainD3 | 0.774820168697353 | 0.163573146724997 |
|  | strainD8 | **0.078358450420483** | **0.126587370532611** |
|  | temperature23:strainD16 | 0.453193338065184 | 0.653253595657988 |
|  | temperature31:strainD16 | 0.285818034403035 | 0.49368569578706 |
|  | temperature23:strainD19 | **0.007259647111238** | **0.022419042159076** |
|  | temperature31:strainD19 | **0.004804122576028** | **0.018379721490756** |
|  | temperature23:strainD21 | 0.119914503486265 | 0.227837556623904 |
|  | temperature31:strainD21 | 0.154548480572862 | 0.21837972149075 |
|  | temperature23:strainD3 | 0.95197820147168 | 0.95197820147168 |
|  | temperature31:strainD3 | 0.704050397818505 | 0.786879856385388 |
|  | temperature23:strainD8 | **0.000178148754267** | **0.001128275443696** |
|  | temperature31:strainD8 | **0.000154889608041** | **0.005214512763986** |
| **Size** | (Intercept) | 4.96114814431928e-06 | 4.57183589746174e-05 |
|  | concentration | **1.229613363451733e-05** | **1.330643243370495e-05** |
|  | temperature23 | **0.001230751306049** | **0.004430704701776** |
|  | temperature31 | **0.001507981766384** | **0.004571835897461** |
|  | strainD16 | 0.489729625454436 | 0.607940224702058 |
|  | strainD19 | **0.016301830363023** | **0.045143530236064** |
|  | strainD21 | 0.449459518395502 | 0.57787652365136 |
|  | strainD3 | 0.175427926529913 | 0.267155973651121 |
|  | strainD8 | **0.084978597171209** | **0.167979051590716** |
|  | concentration:temperature23 | **0.033030646000637** | **0.07927355040153** |
|  | concentration:temperature31 | 0.178103982434081 | 0.267155973651121 |
|  | concentration:strainD16 | **8.16588727519923e-05** | **0.000489953236511954** |
|  | concentration:strainD19 | **1.2511867827568e-05** | **9.00854483584894e-05** |
|  | concentration:strainD21 | **0.025177377514414** | **0.064741827894207** |
|  | concentration:strainD3 | **0.004164523406571** | **0.008992284263657** |
|  | concentration:strainD8 | 0.740501174164046 | 0.784060066761931 |
|  | temperature23:strainD16 | 0.622686917236456 | 0.698245022765668 |
|  | temperature31:strainD16 | 0.571168125771945 | 0.685401750926334 |
|  | temperature23:strainD19 | 0.244613128328106 | 0.338695100761993 |
|  | temperature31:strainD19 | **0.006148918472926** | **0.009446755418778** |
|  | temperature23:strainD21 | 0.134051261194904 | 0.241292270150827 |
|  | temperature31:strainD21 | **0.038469706737583** | **0.071465261326647** |
|  | temperature23:strainD3 | 0.640057937535196 | 0.698245022765668 |
|  | temperature31:strainD3 | 0.775675410144695 | 0.797837564720258 |
|  | temperature23:strainD8 | 0.617658900467146 | 0.698245022765668 |
|  | temperature31:strainD8 | **0.088655610561766** | **0.107979051590716** |
|  | concentration:temperature23:strainD16 | **0.001978969833378** | **0.007915879333512** |
|  | concentration:temperature31:strainD16 | 0.150560907497258 | 0.258104412852442 |
|  | concentration:temperature23:strainD19 | **0.001810458592583** | **0.001931092990471** |
|  | concentration:temperature31:strainD19 | **0.000100972007370** | **0.000405437403316** |
|  | concentration:temperature23:strainD21 | 0.164688615838203 | 0.267155973651121 |
|  | concentration:temperature31:strainD21 | 0.267738648697822 | 0.356984864930429 |
|  | concentration:temperature23:strainD3 | **0.000122938035319** | **0.004128846357514** |
|  | concentration:temperature31:strainD3 | **0.005275947758376** | **0.017266738118324** |
|  | concentration:temperature23:strainD8 | **0.036022988963523** | **0.051051725167928** |
|  | concentration:temperature31:strainD8 | 0.885057188785823 | 0.885057188785823 |
| **Elongation** | (Intercept) | 0.00017001474689987 | 0.0005525479274245 |
|  | concentration | **1.450539594342376e-05** | **1.585701472645089e-05** |
|  | temperature23 | 0.507029518411562 | 0.599216703577301 |
|  | temperature31 | **0.0800658830178921** | **0.100095164359178** |
|  | strainD16 | 0.0116993568700014 | 0.021727377044288 |
|  | strainD19 | 0.907281741091615 | 0.938958041673 |
|  | strainD21 | 0.938958041673 | 0.938958041673 |
|  | strainD3 | **1.15370742693538e-05** | **4.9993988500533e-05** |
|  | strainD8 | **0.000140765440093** | **0.001599014424182** |
|  | concentration:temperature23 | **0.046971254133156** | **0.060417535820691** |
|  | concentration:temperature31 | **0.061796166054601** | **0.093400634839297** |
|  | concentration:strainD16 | 0.625187312678734 | 0.706733483897699 |
|  | concentration:strainD19 | 0.194299668923514 | 0.265883757474283 |
|  | concentration:strainD21 | **0.013963235443475** | **0.0187205887900517** |
|  | concentration:strainD3 | 0.117517127511777 | 0.130095164359178 |
|  | concentration:strainD8 | 0.168982029445862 | 0.488170307288045 |
|  | temperature23:strainD16 | 0.484265307348148 | 0.599216703577301 |
|  | temperature31:strainD16 | **0.034375019021153** | **0.059583366303333** |
|  | temperature23:strainD19 | **0.052614957004121** | **0.081749305131697** |
|  | temperature31:strainD19 | **0.009067100367707** | **0.012134200735414** |
|  | temperature23:strainD21 | **0.004929078502919** | **0.021901300342299** |
|  | temperature31:strainD21 | **1.02645393272484e-5** | **1.93439011254229e-05** |
|  | temperature23:strainD3 | **0.003723833126933** | **0.011675642244372** |
|  | temperature31:strainD3 | **0.004939694795696** | **0.011675642244372** |
|  | temperature23:strainD8 | **1.26171904915988e-05** | **3.42011738195392e-05** |
|  | temperature31:strainD8 | 0.893338622787973 | 0.938958041673 |
| **Velocity** | (Intercept) | 0.814692523934198 | 0.877028835721159 |
|  | concentration | **0.1828305011514428e-05** | **0.008770288357211** |
|  | temperature23 | **0.016072605346815** | **0.096435632080892** |
|  | temperature31 | 0.5604093004502 | 0.681540352083924 |
|  | strainD16 | 0.326921473066675 | 0.511703175234795 |
|  | strainD19 | **0.006067471896553** | **0.010809662758642** |
|  | strainD21 | 0.139428700487433 | 0.358530944110543 |
|  | strainD3 | 0.170029663200944 | 0.405789106176295 |
|  | strainD8 | **0.0807188040742299** | **0.083972001831078** |
|  | concentration:temperature23 | 0.498168815879251 | 0.640502763273323 |
|  | concentration:temperature31 | 0.407261616297509 | 0.543015488396678 |
|  | concentration:strainD16 | 0.987122194448649 | 0.987122194448649 |
|  | concentration:strainD19 | 0.256666465607753 | 0.511703175234795 |
|  | concentration:strainD21 | 0.56795029340327 | 0.681540352083924 |
|  | concentration:strainD3 | **0.0059132633373991** | **0.007438740073184** |
|  | concentration:strainD8 | 0.358648938474192 | 0.516454471402836 |
|  | temperature23:strainD16 | **0.0397459154900599** | **0.053057312628088** |
|  | temperature31:strainD16 | **0.0001237180957511** | **0.007243177886142** |
|  | temperature23:strainD19 | **1.79558011522551e-05** | **6.46408841481183e-05** |
|  | temperature31:strainD19 | **0.00967194417152653** | **0.0780474975437387** |
|  | temperature23:strainD21 | **0.0264264504035397** | **0.035907459218204** |
|  | temperature31:strainD21 | **0.042515920174469** | **0.053057312628088** |
|  | temperature23:strainD3 | **0.0795319377508402** | **0.090397200183107** |
|  | temperature31:strainD3 | **0.0796565562167781** | **0.090397200183107** |
|  | temperature23:strainD8 | 0.316948955890143 | 0.511703175234795 |
|  | temperature31:strainD8 | 0.180350713856131 | 0.405789106176295 |
|  | concentration:temperature23:strainD16 | 0.290055472478688 | 0.511703175234795 |
|  | concentration:temperature31:strainD16 | **0.009375407618044** | **0.0164327640713196** |
|  | concentration:temperature23:strainD19 | 0.750325445397281 | 0.844116126071941 |
|  | concentration:temperature31:strainD19 | 0.357466114659751 | 0.516454471402836 |
|  | concentration:temperature23:strainD21 | 0.29951826022056 | 0.511703175234795 |
|  | concentration:temperature31:strainD21 | **0.0378790583062086** | **0.053057312628088** |
|  | concentration:temperature23:strainD3 | 0.313313992518548 | 0.511703175234795 |
|  | concentration:temperature31:strainD3 | 0.399395954538448 | 0.543015488396678 |
|  | concentration:temperature23:strainD8 | **0.0122436156293423** | **0.0481540325312648** |
|  | concentration:temperature31:strainD8 | 0.197411689948602 | 0.418048284597039 |
| **Accumulation amount** | (Intercept) | 0.47328283721223 | 0.925684308702976 |
|  | concentration | **5.1707275655836e-22** | **1.24097461574006e-20** |
|  | temperature23 | 0.389743936865979 | 0.925684308702976 |
|  | temperature31 | 0.303012985173373 | 0.90903895552012 |
|  | strainD16 | 0.15288716709526 | 0.818083642893114 |
|  | strainD19 | 0.649578663268442 | 0.925684308702976 |
|  | strainD21 | 0.825227212194094 | 0.925684308702976 |
|  | strainD3 | **0.0057742074157597** | **0.00725684308702976** |
|  | strainD8 | **0.0050008627625541** | **0.00725684308702976** |
|  | concentration:strainD16 | 0.650370180474181 | 0.925684308702976 |
|  | concentration:strainD19 | 0.259314718461215 | 0.889079034724167 |
|  | concentration:strainD21 | 0.866937549299587 | 0.925684308702976 |
|  | concentration:strainD3 | **0.034075905442251** | **0.0725684308702976** |
|  | concentration:strainD8 | **0.008279857734646** | **0.0136019868137002** |
|  | temperature23:strainD16 | 0.594520184459352 | 0.925684308702976 |
|  | temperature31:strainD16 | 0.392002483517125 | 0.736019868137002 |
|  | temperature23:strainD19 | 0.925684308702976 | 0.925684308702976 |
|  | temperature31:strainD19 | 0.204520910723278 | 0.818083642893114 |
|  | temperature23:strainD21 | 0.706077949376066 | 0.925684308702976 |
|  | temperature31:strainD21 | 0.74566748181081 | 0.925684308702976 |
|  | temperature23:strainD3 | 0.823968394729309 | 0.925684308702976 |
|  | temperature31:strainD3 | 0.894728338077236 | 0.925684308702976 |
|  | temperature23:strainD8 | 0.611564179236632 | 0.925684308702976 |
|  | temperature31:strainD8 | 0.181318700121912 | 0.818083642893114 |
| **Accumulation percentage** | (Intercept) | 0.12819067095439 | 0.47691440257263 |
|  | strainD16 | 0.116893447517766 | 0.140272137021319 |
|  | strainD19 | 0.00117747802774972 | 0.00176621704162458 |
|  | strainD21 | 0.274180354583115 | 0.274180354583115 |
|  | strainD3 | **0.018108076657566048** | **0.0216153315132096** |
|  | strainD8 | **6.52838847951112e-15** | **1.95851654385334e-14** |

*Figure S5: Effects of Copper concentration gradients on phenotypic traits at different temperatures. The colors represent each of the six strains.* *The points represent the values of each trait at the corresponding copper concentration. The lines are the regressions obtained from the glm models*


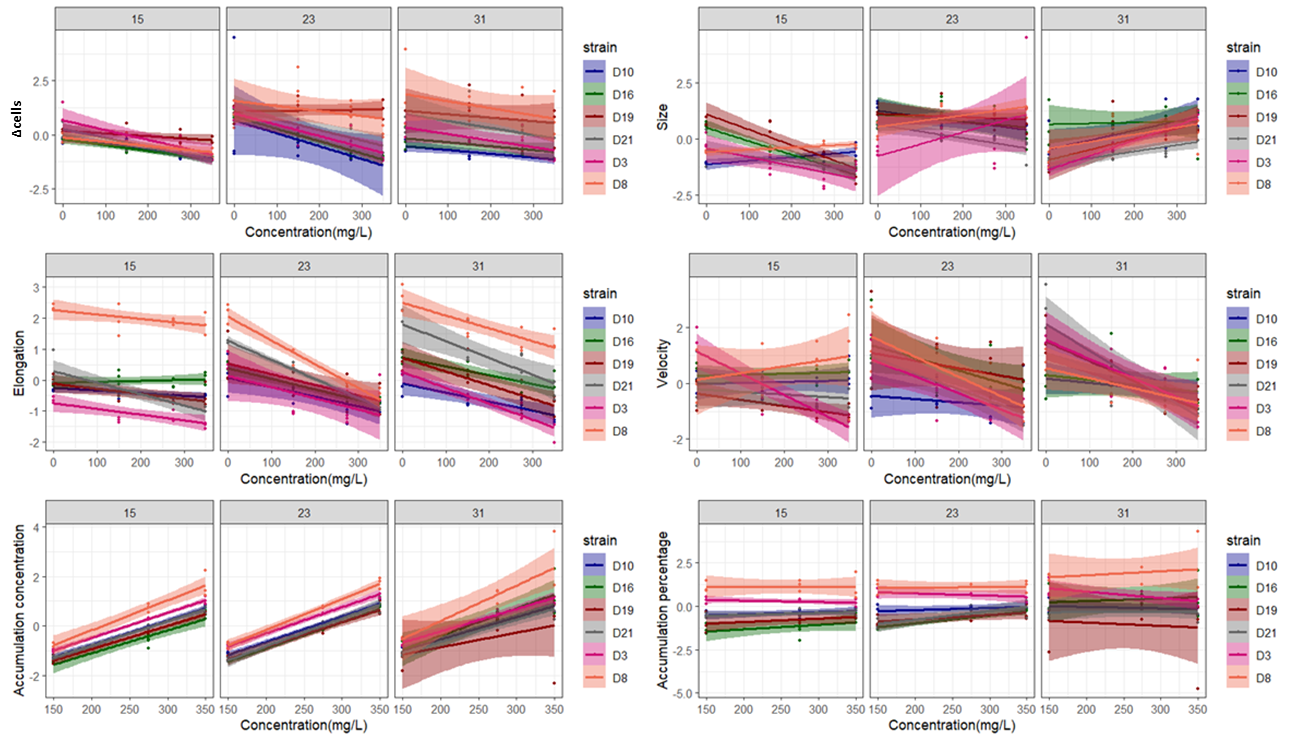


*Figure S6: Schematic representation of effect size of the correlation between accumulation and demography, size, elongation, and swimming velocity for each strain under the three exposure temperatures 15°C, 23°C and 31°C. The points represent effect coefficient and lines represent confidence interval (CI).*


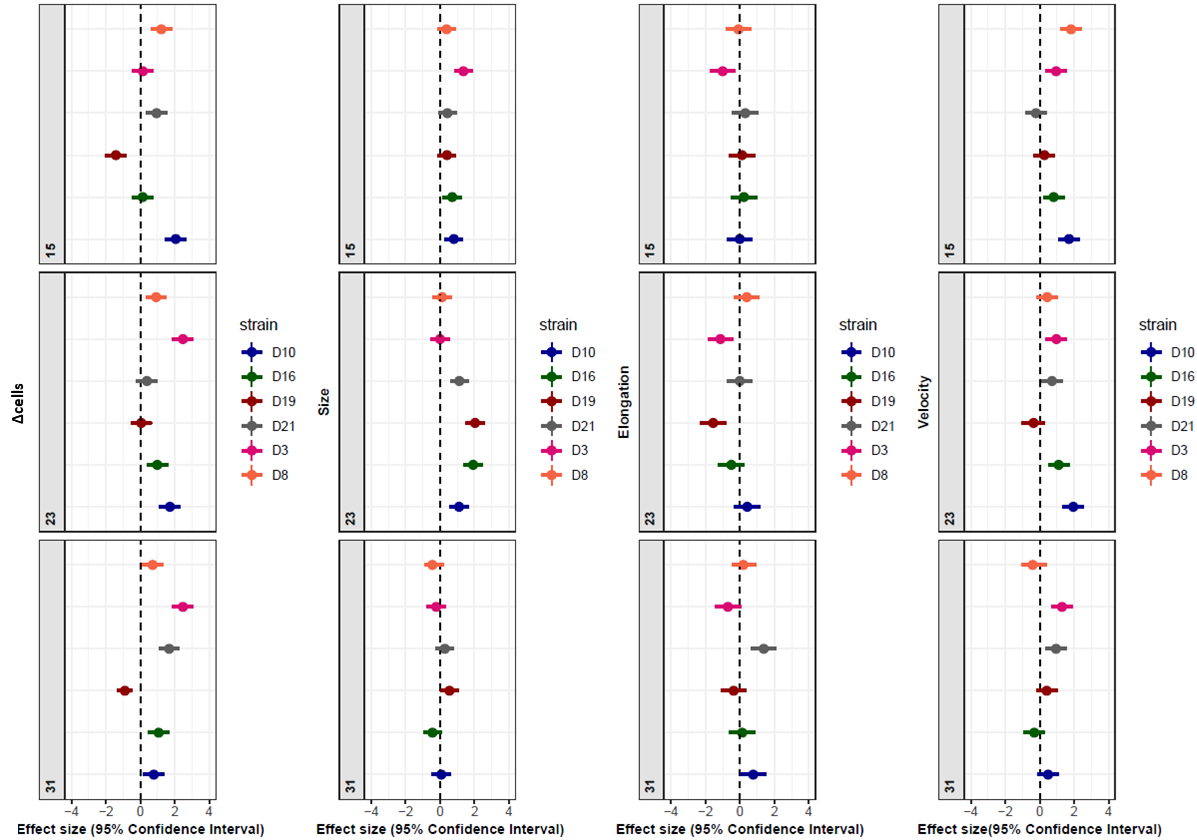


*Table S3:  parameters to assess the quality of fit of SEM model*

| Temperature  (°C) | Statistic | | | | | |
| --- | --- | --- | --- | --- | --- | --- |
|  | **Chi^2^** | **RMSEA** | **SRMR** | **CFI** | ***p*-value** | **AIC** |
| 15 | 175.238 | 0.037 | 0 | 0.879 | 0.23 | 123.871 |
| 23 | 125.612 | 0.009 | 0 | 0.985 | 0.63 | 223.937 |
| 31 | 139.437 | 0.052 | 0 | 0.957 | 0.19 | 316.252 |

*Table S4: Results of SEM models by temperature for each strain (significant effects in red)*

| Label | Estimate | SE | p-val | Confidence Interval | Group | T °C |
| --- | --- | --- | --- | --- | --- | --- |
| elongation.ON.concentration.D10 | -0.5256 | 0.5390 | 0.3295 | [-1.5821, 0.5309] | D10 | 15 |
| elongation.ON.accumulation.D10 | 0.2976 | 0.3775 | 0.4304 | [-0.4422, 1.0375] | D10 | 15 |
| velocity.ON.concentration.D10 | -4.2463* | 1.8805 | 0.0239 | [-7.9320, -0.5605] | D10 | 15 |
| velocity.ON.accumulation.D10 | 3.1472* | 1.3169 | 0.0169 | [0.5662, 5.7282] | D10 | 15 |
| Δcells.ON.concentration.D10 | -3.4022*** | 0.7697 | 0.0000 | [-4.9109, -1.8936] | D10 | 15 |
| Δcells.ON.accumulation.D10 | 1.7033** | 0.5390 | 0.0016 | [0.6469, 2.7598] | D10 | 15 |
| size.ON.concentration.D10 | -0.3786 | 1.1240 | 0.7363 | [-2.5815, 1.8244] | D10 | 15 |
| size.ON.accumulation.D10 | 0.6035 | 0.7871 | 0.4432 | [-0.9391, 2.1461] | D10 | 15 |
| accumulation.ON.concentration.D10 | 1.4171*** | 0.0588 | 0.0000 | [1.3018, 1.5324] | D10 | 15 |
| Variances.elongation.D10 | 0.0152* | 0.0072 | 0.0339 | [0.0012, 0.0293] | D10 | 15 |
| Variances.velocity.D10 | 0.1852* | 0.0873 | 0.0339 | [0.0141, 0.3562] | D10 | 15 |
| Variances.Δcells.D10 | 0.0310* | 0.0146 | 0.0339 | [0.0024, 0.0597] | D10 | 15 |
| Variances.size.D10 | 0.0661* | 0.0312 | 0.0339 | [0.0050, 0.1273] | D10 | 15 |
| Variances.accumulation.D10 | 0.0119* | 0.0056 | 0.0339 | [0.0009, 0.0228] | D10 | 15 |
| Variances.concentration.D10 | 0.3809 | 0.0000 | NA | [0.3809, 0.3809] | D10 | 15 |
| Means.elongation.D10 | -0.1087 | 0.2937 | 0.7114 | [-0.6842, 0.4669] | D10 | 15 |
| Means.velocity.D10 | 2.4635* | 1.0245 | 0.0162 | [0.4555, 4.4716] | D10 | 15 |
| Means.Δcells.D10 | 1.0937** | 0.4193 | 0.0091 | [0.2718, 1.9156] | D10 | 15 |
| Means.size.D10 | 0.1843 | 0.6123 | 0.7634 | [-1.0159, 1.3845] | D10 | 15 |
| Means.accumulation.D10 | -0.7656*** | 0.0461 | 0.0000 | [-0.8560, -0.6752] | D10 | 15 |
| Means.concentration.D10 | 0.4832 | 0.0000 | NA | [0.4832, 0.4832] | D10 | 15 |
| elongation.ON.concentration.D16 | -0.7223** | 0.2533 | 0.0044 | [-1.2188, -0.2258] | D16 | 15 |
| elongation.ON.accumulation.D16 | 0.5192** | 0.1829 | 0.0045 | [0.1607, 0.8776] | D16 | 15 |
| velocity.ON.concentration.D16 | -0.3593 | 0.4111 | 0.3821 | [-1.1652, 0.4465] | D16 | 15 |
| velocity.ON.accumulation.D16 | 0.5744 | 0.2968 | 0.0530 | [-0.0073, 1.1561] | D16 | 15 |
| Δcells.ON.concentration.D16 | 0.1455 | 0.1646 | 0.3770 | [-0.1772, 0.4681] | D16 | 15 |
| Δcells.ON.accumulation.D16 | -0.3929*** | 0.1188 | 0.0009 | [-0.6258, -0.1600] | D16 | 15 |
| size.ON.concentration.D16 | -1.3045* | 0.5645 | 0.0208 | [-2.4109, -0.1981] | D16 | 15 |
| size.ON.accumulation.D16 | 0.4499 | 0.4075 | 0.2696 | [-0.3488, 1.2485] | D16 | 15 |
| accumulation.ON.concentration.D16 | 1.3245*** | 0.1353 | 0.0000 | [1.0594, 1.5896] | D16 | 15 |
| Variances.elongation.D16 | 0.0189* | 0.0089 | 0.0339 | [0.0014, 0.0363] | D16 | 15 |
| Variances.velocity.D16 | 0.0497* | 0.0234 | 0.0339 | [0.0038, 0.0957] | D16 | 15 |
| Variances.Δcells.D16 | 0.0080* | 0.0038 | 0.0339 | [0.0006, 0.0153] | D16 | 15 |
| Variances.size.D16 | 0.0937* | 0.0442 | 0.0339 | [0.0071, 0.1803] | D16 | 15 |
| Variances.accumulation.D16 | 0.0627* | 0.0296 | 0.0339 | [0.0048, 0.1207] | D16 | 15 |
| Variances.concentration.D16 | 0.3809 | 0.0000 | NA | [0.3809, 0.3809] | D16 | 15 |
| Means.elongation.D16 | 0.6732** | 0.2118 | 0.0015 | [0.2580, 1.0884] | D16 | 15 |
| Means.velocity.D16 | 0.9011** | 0.3438 | 0.0088 | [0.2273, 1.5750] | D16 | 15 |
| Means.Δcells.D16 | -1.1276*** | 0.1377 | 0.0000 | [-1.3974, -0.8577] | D16 | 15 |
| Means.size.D16 | 0.3287 | 0.4720 | 0.4863 | [-0.5965, 1.2538] | D16 | 15 |
| Means.accumulation.D16 | -1.1138*** | 0.1060 | 0.0000 | [-1.3216, -0.9060] | D16 | 15 |
| Means.concentration.D16 | 0.4832 | 0.0000 | NA | [0.4832, 0.4832] | D16 | 15 |
| elongation.ON.concentration.D19 | -0.5488 | 0.7314 | 0.4530 | [-1.9823, 0.8847] | D19 | 15 |
| elongation.ON.accumulation.D19 | 0.4168 | 0.5391 | 0.4394 | [-0.6398, 1.4733] | D19 | 15 |
| velocity.ON.concentration.D19 | 0.0275 | 1.0988 | 0.9800 | [-2.1260, 2.1811] | D19 | 15 |
| velocity.ON.accumulation.D19 | -0.1538 | 0.8098 | 0.8494 | [-1.7411, 1.4335] | D19 | 15 |
| Δcells.ON.concentration.D19 | 2.3472 | 1.5640 | 0.1334 | [-0.7182, 5.4126] | D19 | 15 |
| Δcells.ON.accumulation.D19 | -2.0886 | 1.1527 | 0.0700 | [-4.3479, 0.1708] | D19 | 15 |
| size.ON.concentration.D19 | -1.9771 | 1.1672 | 0.0903 | [-4.2648, 0.3107] | D19 | 15 |
| size.ON.accumulation.D19 | -0.0380 | 0.8603 | 0.9648 | [-1.7241, 1.6482] | D19 | 15 |
| accumulation.ON.concentration.D19 | 1.3420*** | 0.0665 | 0.0000 | [1.2118, 1.4723] | D19 | 15 |
| Variances.elongation.D19 | 0.0396* | 0.0187 | 0.0339 | [0.0030, 0.0762] | D19 | 15 |
| Variances.velocity.D19 | 0.0894* | 0.0422 | 0.0339 | [0.0068, 0.1720] | D19 | 15 |
| Variances.Δcells.D19 | 0.1812* | 0.0854 | 0.0339 | [0.0138, 0.3485] | D19 | 15 |
| Variances.size.D19 | 0.1009* | 0.0476 | 0.0339 | [0.0077, 0.1941] | D19 | 15 |
| Variances.accumulation.D19 | 0.0151* | 0.0071 | 0.0339 | [0.0012, 0.0291] | D19 | 15 |
| Variances.concentration.D19 | 0.3809 | 0.0000 | NA | [0.3809, 0.3809] | D19 | 15 |
| Means.elongation.D19 | -0.1109 | 0.5093 | 0.8277 | [-1.1091, 0.8874] | D19 | 15 |
| Means.velocity.D19 | -1.0432 | 0.7652 | 0.1728 | [-2.5429, 0.4565] | D19 | 15 |
| Means.Δcells.D19 | -1.1318 | 1.0892 | 0.2988 | [-3.2665, 1.0030] | D19 | 15 |
| Means.size.D19 | 1.1647 | 0.8129 | 0.1519 | [-0.4285, 2.7579] | D19 | 15 |
| Means.accumulation.D19 | -0.9318*** | 0.0521 | 0.0000 | [-1.0340, -0.8297] | D19 | 15 |
| Means.concentration.D19 | 0.4832 | 0.0000 | NA | [0.4832, 0.4832] | D19 | 15 |
| elongation.ON.concentration.D21 | -1.2165 | 0.8536 | 0.1541 | [-2.8895, 0.4564] | D21 | 15 |
| elongation.ON.accumulation.D21 | 0.5902 | 0.6047 | 0.3291 | [-0.5950, 1.7753] | D21 | 15 |
| velocity.ON.concentration.D21 | 0.4191 | 2.2273 | 0.8507 | [-3.9463, 4.7845] | D21 | 15 |
| velocity.ON.accumulation.D21 | -0.1966 | 1.5779 | 0.9008 | [-3.2892, 2.8960] | D21 | 15 |
| Δcells.ON.concentration.D21 | 0.4850 | 1.1378 | 0.6699 | [-1.7450, 2.7151] | D21 | 15 |
| Δcells.ON.accumulation.D21 | -0.8244 | 0.8061 | 0.3064 | [-2.4042, 0.7555] | D21 | 15 |
| size.ON.concentration.D21 | -0.4249 | 0.7804 | 0.5862 | [-1.9545, 1.1048] | D21 | 15 |
| size.ON.accumulation.D21 | 0.0024 | 0.5529 | 0.9965 | [-1.0812, 1.0861] | D21 | 15 |
| accumulation.ON.concentration.D21 | 1.4022*** | 0.0542 | 0.0000 | [1.2960, 1.5083] | D21 | 15 |
| Variances.elongation.D21 | 0.0331* | 0.0156 | 0.0339 | [0.0025, 0.0637] | D21 | 15 |
| Variances.velocity.D21 | 0.2254* | 0.1063 | 0.0339 | [0.0171, 0.4337] | D21 | 15 |
| Variances.Δcells.D21 | 0.0588* | 0.0277 | 0.0339 | [0.0045, 0.1132] | D21 | 15 |
| Variances.size.D21 | 0.0277* | 0.0130 | 0.0339 | [0.0021, 0.0532] | D21 | 15 |
| Variances.accumulation.D21 | 0.0101* | 0.0047 | 0.0339 | [0.0008, 0.0194] | D21 | 15 |
| Variances.concentration.D21 | 0.3809 | 0.0000 | NA | [0.3809, 0.3809] | D21 | 15 |
| Means.elongation.D21 | 0.0628 | 0.4818 | 0.8962 | [-0.8815, 1.0071] | D21 | 15 |
| Means.velocity.D21 | -0.6861 | 1.2572 | 0.5852 | [-3.1501, 1.7779] | D21 | 15 |
| Means.Δcells.D21 | -1.0052 | 0.6422 | 0.1175 | [-2.2639, 0.2535] | D21 | 15 |
| Means.size.D21 | -0.4419 | 0.4405 | 0.3158 | [-1.3053, 0.4215] | D21 | 15 |
| Means.accumulation.D21 | -0.7865*** | 0.0425 | 0.0000 | [-0.8697, -0.7033] | D21 | 15 |
| Means.concentration.D21 | 0.4832 | 0.0000 | NA | [0.4832, 0.4832] | D21 | 15 |
| elongation.ON.concentration.D3 | 0.8255 | 1.2855 | 0.5208 | [-1.6939, 3.3449] | D3 | 15 |
| elongation.ON.accumulation.D3 | -0.6159 | 0.8788 | 0.4834 | [-2.3382, 1.1064] | D3 | 15 |
| velocity.ON.concentration.D3 | -1.5415 | 1.6448 | 0.3487 | [-4.7651, 1.6822] | D3 | 15 |
| velocity.ON.accumulation.D3 | 0.7678 | 1.1244 | 0.4947 | [-1.4359, 2.9715] | D3 | 15 |
| Δcells.ON.concentration.D3 | -0.7444 | 2.8417 | 0.7934 | [-6.3140, 4.8252] | D3 | 15 |
| Δcells.ON.accumulation.D3 | -0.3795 | 1.9426 | 0.8451 | [-4.1870, 3.4280] | D3 | 15 |
| size.ON.concentration.D3 | -2.1496 | 3.1157 | 0.4903 | [-8.2563, 3.9571] | D3 | 15 |
| size.ON.accumulation.D3 | 1.4856 | 2.1300 | 0.4855 | [-2.6891, 5.6602] | D3 | 15 |
| accumulation.ON.concentration.D3 | 1.4564*** | 0.0458 | 0.0000 | [1.3667, 1.5460] | D3 | 15 |
| Variances.elongation.D3 | 0.0499* | 0.0235 | 0.0339 | [0.0038, 0.0960] | D3 | 15 |
| Variances.velocity.D3 | 0.0817* | 0.0385 | 0.0339 | [0.0062, 0.1571] | D3 | 15 |
| Variances.Δcells.D3 | 0.2438* | 0.1149 | 0.0339 | [0.0185, 0.4690] | D3 | 15 |
| Variances.size.D3 | 0.2931* | 0.1382 | 0.0339 | [0.0223, 0.5638] | D3 | 15 |
| Variances.accumulation.D3 | 0.0072* | 0.0034 | 0.0339 | [0.0005, 0.0138] | D3 | 15 |
| Variances.concentration.D3 | 0.3809 | 0.0000 | NA | [0.3809, 0.3809] | D3 | 15 |
| Means.elongation.D3 | -1.3872*** | 0.4121 | 0.0008 | [-2.1948, -0.5796] | D3 | 15 |
| Means.velocity.D3 | -0.4629 | 0.5272 | 0.3800 | [-1.4962, 0.5705] | D3 | 15 |
| Means.Δcells.D3 | 0.4044 | 0.9109 | 0.6571 | [-1.3809, 2.1898] | D3 | 15 |
| Means.size.D3 | -0.4136 | 0.9987 | 0.6788 | [-2.3711, 1.5439] | D3 | 15 |
| Means.accumulation.D3 | -0.4564*** | 0.0359 | 0.0000 | [-0.5267, -0.3861] | D3 | 15 |
| Means.concentration.D3 | 0.4832 | 0.0000 | NA | [0.4832, 0.4832] | D3 | 15 |
| elongation.ON.concentration.D8 | -0.4631 | 0.5818 | 0.4260 | [-1.6034, 0.6771] | D8 | 15 |
| elongation.ON.accumulation.D8 | 0.2268 | 0.3236 | 0.4834 | [-0.4074, 0.8609] | D8 | 15 |
| velocity.ON.concentration.D8 | -2.9729 | 2.1997 | 0.1765 | [-7.2842, 1.3384] | D8 | 15 |
| velocity.ON.accumulation.D8 | 1.9467 | 1.2234 | 0.1116 | [-0.4512, 4.3445] | D8 | 15 |
| Δcells.ON.concentration.D8 | -2.1224*** | 0.5144 | 0.0000 | [-3.1306, -1.1142] | D8 | 15 |
| Δcells.ON.accumulation.D8 | 0.7844** | 0.2861 | 0.0061 | [0.2236, 1.3451] | D8 | 15 |
| size.ON.concentration.D8 | 0.3097 | 0.4601 | 0.5009 | [-0.5921, 1.2114] | D8 | 15 |
| size.ON.accumulation.D8 | -0.0763 | 0.2559 | 0.7657 | [-0.5778, 0.4253] | D8 | 15 |
| accumulation.ON.concentration.D8 | 1.7365*** | 0.1554 | 0.0000 | [1.4319, 2.0411] | D8 | 15 |
| Variances.elongation.D8 | 0.0780* | 0.0368 | 0.0339 | [0.0059, 0.1501] | D8 | 15 |
| Variances.velocity.D8 | 1.1156* | 0.5259 | 0.0339 | [0.0849, 2.1463] | D8 | 15 |
| Variances.Δcells.D8 | 0.0610* | 0.0288 | 0.0339 | [0.0046, 0.1174] | D8 | 15 |
| Variances.size.D8 | 0.0488* | 0.0230 | 0.0339 | [0.0037, 0.0939] | D8 | 15 |
| Variances.accumulation.D8 | 0.0828* | 0.0390 | 0.0339 | [0.0063, 0.1593] | D8 | 15 |
| Variances.concentration.D8 | 0.3809 | 0.0000 | NA | [0.3809, 0.3809] | D8 | 15 |
| Means.elongation.D8 | 1.9318*** | 0.1265 | 0.0000 | [1.6838, 2.1798] | D8 | 15 |
| Means.velocity.D8 | 1.0198* | 0.4785 | 0.0331 | [0.0820, 1.9576] | D8 | 15 |
| Means.Δcells.D8 | 0.2071 | 0.1119 | 0.0642 | [-0.0122, 0.4264] | D8 | 15 |
| Means.size.D8 | 0.4202*** | 0.1001 | 0.0000 | [0.2240, 0.6163] | D8 | 15 |
| Means.accumulation.D8 | -0.1392 | 0.1218 | 0.2531 | [-0.3780, 0.0995] | D8 | 15 |
| Means.concentration.D8 | 0.4832 | 0.0000 | NA | [0.4832, 0.4832] | D8 | 15 |

| Label | Estimate | SE | pval | Confidence Interval | Group | T °C |
| --- | --- | --- | --- | --- | --- | --- |
| elongation.ON.concentration.D10 | -1.3252 | 1.6937 | 0.4340 | [-4.6448, 1.9945] | D10 | 23 |
| elongation.ON.accumulation.D10 | 0.6988 | 1.1748 | 0.5520 | [-1.6039, 3.0014] | D10 | 23 |
| velocity.ON.concentration.D10 | -5.2285* | 2.6230 | 0.0462 | [-10.3695, -0.0876] | D10 | 23 |
| velocity.ON.accumulation.D10 | 3.4822 | 1.8194 | 0.0556 | [-0.0837, 7.0482] | D10 | 23 |
| Δcells.ON.concentration.D10 | -2.2054*** | 0.4042 | 0.0000 | [-2.9976, -1.4132] | D10 | 23 |
| Δcells.ON.accumulation.D10 | 1.3319*** | 0.2804 | 0.0000 | [0.7824, 1.8814] | D10 | 23 |
| size.ON.concentration.D10 | -1.3577 | 2.0244 | 0.5024 | [-5.3255, 2.6100] | D10 | 23 |
| size.ON.accumulation.D10 | 1.0827 | 1.4042 | 0.4407 | [-1.6695, 3.8349] | D10 | 23 |
| accumulation.ON.concentration.D10 | 1.4329*** | 0.0529 | 0.0000 | [1.3292, 1.5366] | D10 | 23 |
| Variances.elongation.D10 | 0.1192* | 0.0562 | 0.0339 | [0.0091, 0.2294] | D10 | 23 |
| Variances.velocity.D10 | 0.2860* | 0.1348 | 0.0339 | [0.0218, 0.5502] | D10 | 23 |
| Variances.Δcells.D10 | 0.0068* | 0.0032 | 0.0339 | [0.0005, 0.0131] | D10 | 23 |
| Variances.size.D10 | 0.1703* | 0.0803 | 0.0339 | [0.0130, 0.3277] | D10 | 23 |
| Variances.accumulation.D10 | 0.0096* | 0.0045 | 0.0339 | [0.0007, 0.0185] | D10 | 23 |
| Variances.concentration.D10 | 0.3809 | 0.0000 | NA | [0.3809, 0.3809] | D10 | 23 |
| Means.elongation.D10 | -0.0377 | 0.9041 | 0.9668 | [-1.8096, 1.7343] | D10 | 23 |
| Means.velocity.D10 | 2.0610 | 1.4000 | 0.1410 | [-0.6830, 4.8050] | D10 | 23 |
| Means.Δcells.D10 | 0.0513 | 0.2157 | 0.8121 | [-0.3716, 0.4741] | D10 | 23 |
| Means.size.D10 | 0.5909 | 1.0806 | 0.5845 | [-1.5269, 2.7088] | D10 | 23 |
| Means.accumulation.D10 | -0.7594*** | 0.0415 | 0.0000 | [-0.8407, -0.6781] | D10 | 23 |
| Means.concentration.D10 | 0.4832 | 0.0000 | NA | [0.4832, 0.4832] | D10 | 23 |
| elongation.ON.concentration.D16 | -0.0888 | 0.7098 | 0.9004 | [-1.4800, 1.3024] | D16 | 23 |
| elongation.ON.accumulation.D16 | -0.1517 | 0.4807 | 0.7523 | [-1.0940, 0.7905] | D16 | 23 |
| velocity.ON.concentration.D16 | -1.2758 | 2.4115 | 0.5968 | [-6.0023, 3.4508] | D16 | 23 |
| velocity.ON.accumulation.D16 | 0.9710 | 1.6333 | 0.5522 | [-2.2301, 4.1722] | D16 | 23 |
| Δcells.ON.concentration.D16 | -1.3309 | 0.8431 | 0.1144 | [-2.9834, 0.3216] | D16 | 23 |
| Δcells.ON.accumulation.D16 | 0.5320 | 0.5710 | 0.3516 | [-0.5872, 1.6511] | D16 | 23 |
| size.ON.concentration.D16 | -4.6819* | 2.1253 | 0.0276 | [-8.8475, -0.5164] | D16 | 23 |
| size.ON.accumulation.D16 | 2.3855 | 1.4394 | 0.0975 | [-0.4357, 5.2067] | D16 | 23 |
| accumulation.ON.concentration.D16 | 1.4690*** | 0.0497 | 0.0000 | [1.3717, 1.5663] | D16 | 23 |
| Variances.elongation.D16 | 0.0176* | 0.0083 | 0.0339 | [0.0013, 0.0338] | D16 | 23 |
| Variances.velocity.D16 | 0.2030* | 0.0957 | 0.0339 | [0.0154, 0.3905] | D16 | 23 |
| Variances.Δcells.D16 | 0.0248* | 0.0117 | 0.0339 | [0.0019, 0.0477] | D16 | 23 |
| Variances.size.D16 | 0.1576* | 0.0743 | 0.0339 | [0.0120, 0.3033] | D16 | 23 |
| Variances.accumulation.D16 | 0.0085* | 0.0040 | 0.0339 | [0.0006, 0.0163] | D16 | 23 |
| Variances.concentration.D16 | 0.3809 | 0.0000 | NA | [0.3809, 0.3809] | D16 | 23 |
| Means.elongation.D16 | -0.4862 | 0.4947 | 0.3256 | [-1.4558, 0.4833] | D16 | 23 |
| Means.velocity.D16 | 0.9187 | 1.6806 | 0.5846 | [-2.3752, 4.2126] | D16 | 23 |
| Means.Δcells.D16 | -0.0223 | 0.5876 | 0.9697 | [-1.1739, 1.1293] | D16 | 23 |
| Means.size.D16 | 3.2196* | 1.4811 | 0.0297 | [0.3167, 6.1226] | D16 | 23 |
| Means.accumulation.D16 | -1.0223*** | 0.0389 | 0.0000 | [-1.0986, -0.9460] | D16 | 23 |
| Means.concentration.D16 | 0.4832 | 0.0000 | NA | [0.4832, 0.4832] | D16 | 23 |
| elongation.ON.concentration.D19 | 2.3355* | 0.9708 | 0.0161 | [0.4328, 4.2381] | D19 | 23 |
| elongation.ON.accumulation.D19 | -2.0457** | 0.6872 | 0.0029 | [-3.3927, -0.6987] | D19 | 23 |
| velocity.ON.concentration.D19 | 1.9983 | 1.9455 | 0.3044 | [-1.8149, 5.8115] | D19 | 23 |
| velocity.ON.accumulation.D19 | -1.6915 | 1.3773 | 0.2194 | [-4.3910, 1.0080] | D19 | 23 |
| Δcells.ON.concentration.D19 | 0.5006 | 0.7035 | 0.4767 | [-0.8782, 1.8795] | D19 | 23 |
| Δcells.ON.accumulation.D19 | -0.4841 | 0.4980 | 0.3311 | [-1.4602, 0.4921] | D19 | 23 |
| size.ON.concentration.D19 | -4.0192*** | 0.5991 | 0.0000 | [-5.1935, -2.8450] | D19 | 23 |
| size.ON.accumulation.D19 | 2.5674*** | 0.4241 | 0.0000 | [1.7361, 3.3987] | D19 | 23 |
| accumulation.ON.concentration.D19 | 1.3840*** | 0.0941 | 0.0000 | [1.1995, 1.5685] | D19 | 23 |
| Variances.elongation.D19 | 0.1292* | 0.0609 | 0.0339 | [0.0098, 0.2485] | D19 | 23 |
| Variances.velocity.D19 | 0.5188* | 0.2446 | 0.0339 | [0.0395, 0.9981] | D19 | 23 |
| Variances.Δcells.D19 | 0.0678* | 0.0320 | 0.0339 | [0.0052, 0.1305] | D19 | 23 |
| Variances.size.D19 | 0.0492* | 0.0232 | 0.0339 | [0.0037, 0.0946] | D19 | 23 |
| Variances.accumulation.D19 | 0.0304* | 0.0143 | 0.0339 | [0.0023, 0.0585] | D19 | 23 |
| Variances.concentration.D19 | 0.3809 | 0.0000 | NA | [0.3809, 0.3809] | D19 | 23 |
| Means.elongation.D19 | -1.9843** | 0.6731 | 0.0032 | [-3.3035, -0.6651] | D19 | 23 |
| Means.velocity.D19 | -1.1416 | 1.3489 | 0.3974 | [-3.7855, 1.5022] | D19 | 23 |
| Means.Δcells.D19 | 0.4239 | 0.4878 | 0.3848 | [-0.5321, 1.3800] | D19 | 23 |
| Means.size.D19 | 3.0696*** | 0.4154 | 0.0000 | [2.2554, 3.8837] | D19 | 23 |
| Means.accumulation.D19 | -0.9540*** | 0.0738 | 0.0000 | [-1.0987, -0.8094] | D19 | 23 |
| Means.concentration.D19 | 0.4832 | 0.0000 | NA | [0.4832, 0.4832] | D19 | 23 |
| elongation.ON.concentration.D21 | -1.4558*** | 0.4185 | 0.0005 | [-2.2761, -0.6355] | D21 | 23 |
| elongation.ON.accumulation.D21 | 0.2979 | 0.2644 | 0.2599 | [-0.2203, 0.8161] | D21 | 23 |
| velocity.ON.concentration.D21 | -2.0474* | 0.9335 | 0.0283 | [-3.8769, -0.2178] | D21 | 23 |
| velocity.ON.accumulation.D21 | 0.4420 | 0.5897 | 0.4536 | [-0.7138, 1.5977] | D21 | 23 |
| Δcells.ON.concentration.D21 | -0.7592 | 1.2259 | 0.5357 | [-3.1619, 1.6435] | D21 | 23 |
| Δcells.ON.accumulation.D21 | -0.1275 | 0.7744 | 0.8692 | [-1.6454, 1.3903] | D21 | 23 |
| size.ON.concentration.D21 | -2.1678* | 0.9196 | 0.0184 | [-3.9701, -0.3655] | D21 | 23 |
| size.ON.accumulation.D21 | 1.1083 | 0.5809 | 0.0564 | [-0.0303, 2.2469] | D21 | 23 |
| accumulation.ON.concentration.D21 | 1.5544*** | 0.0998 | 0.0000 | [1.3587, 1.7500] | D21 | 23 |
| Variances.elongation.D21 | 0.0215* | 0.0101 | 0.0339 | [0.0016, 0.0414] | D21 | 23 |
| Variances.velocity.D21 | 0.1069* | 0.0504 | 0.0339 | [0.0081, 0.2058] | D21 | 23 |
| Variances.Δcells.D21 | 0.1844* | 0.0869 | 0.0339 | [0.0140, 0.3549] | D21 | 23 |
| Variances.size.D21 | 0.1038* | 0.0489 | 0.0339 | [0.0079, 0.1997] | D21 | 23 |
| Variances.accumulation.D21 | 0.0342* | 0.0161 | 0.0339 | [0.0026, 0.0657] | D21 | 23 |
| Variances.concentration.D21 | 0.3809 | 0.0000 | NA | [0.3809, 0.3809] | D21 | 23 |
| Means.elongation.D21 | 0.5366* | 0.2560 | 0.0361 | [0.0348, 1.0383] | D21 | 23 |
| Means.velocity.D21 | 0.6678 | 0.5709 | 0.2421 | [-0.4511, 1.7868] | D21 | 23 |
| Means.Δcells.D21 | 0.1094 | 0.7498 | 0.8839 | [-1.3601, 1.5790] | D21 | 23 |
| Means.size.D21 | 0.3600 | 0.5624 | 0.5221 | [-0.7423, 1.4623] | D21 | 23 |
| Means.accumulation.D21 | -0.9393*** | 0.0783 | 0.0000 | [-1.0926, -0.7859] | D21 | 23 |
| Means.concentration.D21 | 0.4832 | 0.0000 | NA | [0.4832, 0.4832] | D21 | 23 |
| elongation.ON.concentration.D3 | 2.8796 | 1.8975 | 0.1291 | [-0.8393, 6.5986] | D3 | 23 |
| elongation.ON.accumulation.D3 | -1.6565 | 1.2847 | 0.1973 | [-4.1743, 0.8614] | D3 | 23 |
| velocity.ON.concentration.D3 | -1.3566 | 1.9169 | 0.4791 | [-5.1136, 2.4004] | D3 | 23 |
| velocity.ON.accumulation.D3 | 0.7887 | 1.2978 | 0.5434 | [-1.7550, 3.3323] | D3 | 23 |
| Δcells.ON.concentration.D3 | -3.8373* | 1.4903 | 0.0100 | [-6.7582, -0.9164] | D3 | 23 |
| Δcells.ON.accumulation.D3 | 2.1519* | 1.0090 | 0.0329 | [0.1743, 4.1295] | D3 | 23 |
| size.ON.concentration.D3 | 7.9277 | 7.9069 | 0.3160 | [-7.5695, 23.4249] | D3 | 23 |
| size.ON.accumulation.D3 | -4.5117 | 5.3533 | 0.3993 | [-15.0040, 5.9805] | D3 | 23 |
| accumulation.ON.concentration.D3 | 1.4647*** | 0.0672 | 0.0000 | [1.3330, 1.5965] | D3 | 23 |
| Variances.elongation.D3 | 0.1729* | 0.0864 | 0.0455 | [0.0035, 0.3423] | D3 | 23 |
| Variances.velocity.D3 | 0.1764* | 0.0882 | 0.0455 | [0.0035, 0.3494] | D3 | 23 |
| Variances.Δcells.D3 | 0.1067* | 0.0533 | 0.0455 | [0.0021, 0.2112] | D3 | 23 |
| Variances.size.D3 | 3.0022* | 1.5011 | 0.0455 | [0.0601, 5.9443] | D3 | 23 |
| Variances.accumulation.D3 | 0.0131* | 0.0065 | 0.0455 | [0.0003, 0.0259] | D3 | 23 |
| Variances.concentration.D3 | 0.3624 | 0.0000 | NA | [0.3624, 0.3624] | D3 | 23 |
| Means.elongation.D3 | -1.7766*** | 0.5368 | 0.0009 | [-2.8286, -0.7245] | D3 | 23 |
| Means.velocity.D3 | -0.3170 | 0.5423 | 0.5589 | [-1.3798, 0.7459] | D3 | 23 |
| Means.Δcells.D3 | 0.6167 | 0.4216 | 0.1435 | [-0.2096, 1.4430] | D3 | 23 |
| Means.size.D3 | -2.5005 | 2.2368 | 0.2636 | [-6.8845, 1.8835] | D3 | 23 |
| Means.accumulation.D3 | -0.3947*** | 0.0485 | 0.0000 | [-0.4897, -0.2997] | D3 | 23 |
| Means.concentration.D3 | 0.3975 | 0.0000 | NA | [0.3975, 0.3975] | D3 | 23 |
| elongation.ON.concentration.D8 | -2.1422** | 0.6950 | 0.0021 | [-3.5045, -0.7800] | D8 | 23 |
| elongation.ON.accumulation.D8 | 0.6705 | 0.4019 | 0.0952 | [-0.1172, 1.4583] | D8 | 23 |
| velocity.ON.concentration.D8 | -1.3451 | 1.6641 | 0.4189 | [-4.6067, 1.9165] | D8 | 23 |
| velocity.ON.accumulation.D8 | 0.0615 | 0.9623 | 0.9491 | [-1.8245, 1.9475] | D8 | 23 |
| Δcells.ON.concentration.D8 | -2.0015 | 1.3754 | 0.1456 | [-4.6972, 0.6942] | D8 | 23 |
| Δcells.ON.accumulation.D8 | 0.4595 | 0.7953 | 0.5634 | [-1.0993, 2.0183] | D8 | 23 |
| size.ON.concentration.D8 | 0.7035 | 0.5710 | 0.2179 | [-0.4157, 1.8227] | D8 | 23 |
| size.ON.accumulation.D8 | -0.4877 | 0.3302 | 0.1396 | [-1.1349, 0.1594] | D8 | 23 |
| accumulation.ON.concentration.D8 | 1.7073*** | 0.0917 | 0.0000 | [1.5277, 1.8870] | D8 | 23 |
| Variances.elongation.D8 | 0.0419* | 0.0197 | 0.0339 | [0.0032, 0.0806] | D8 | 23 |
| Variances.velocity.D8 | 0.2401* | 0.1132 | 0.0339 | [0.0183, 0.4620] | D8 | 23 |
| Variances.Δcells.D8 | 0.1640* | 0.0773 | 0.0339 | [0.0125, 0.3156] | D8 | 23 |
| Variances.size.D8 | 0.0283* | 0.0133 | 0.0339 | [0.0022, 0.0544] | D8 | 23 |
| Variances.accumulation.D8 | 0.0288* | 0.0136 | 0.0339 | [0.0022, 0.0554] | D8 | 23 |
| Variances.concentration.D8 | 0.3809 | 0.0000 | NA | [0.3809, 0.3809] | D8 | 23 |
| Means.elongation.D8 | 0.8511*** | 0.1379 | 0.0000 | [0.5808, 1.1214] | D8 | 23 |
| Means.velocity.D8 | 0.4907 | 0.3302 | 0.1373 | [-0.1565, 1.1379] | D8 | 23 |
| Means.Δcells.D8 | 1.4986*** | 0.2729 | 0.0000 | [0.9637, 2.0335] | D8 | 23 |
| Means.size.D8 | 0.6773*** | 0.1133 | 0.0000 | [0.4553, 0.8994] | D8 | 23 |
| Means.accumulation.D8 | -0.2670*** | 0.0719 | 0.0002 | [-0.4078, -0.1262] | D8 | 23 |
| Means.concentration.D8 | 0.4832 | 0.0000 | NA | [0.4832, 0.4832] | D8 | 23 |

| Label | Estimate | SE | pval | Confidence Interval | Group | T °C |
| --- | --- | --- | --- | --- | --- | --- |
| elongation.ON.concentration.D10 | -1.6108* | 0.8097 | 0.0467 | [-3.1977, -0.0238] | D10 | 31 |
| elongation.ON.accumulation.D10 | 1.0162 | 0.7191 | 0.1576 | [-0.3931, 2.4255] | D10 | 31 |
| velocity.ON.concentration.D10 | -0.6970 | 0.8630 | 0.4193 | [-2.3884, 0.9944] | D10 | 31 |
| velocity.ON.accumulation.D10 | 0.1310 | 0.7664 | 0.8643 | [-1.3711, 1.6331] | D10 | 31 |
| Δcells.ON.concentration.D10 | -0.3818 | 0.4354 | 0.3806 | [-1.2353, 0.4716] | D10 | 31 |
| Δcells.ON.accumulation.D10 | 0.3104 | 0.3867 | 0.4222 | [-0.4475, 1.0683] | D10 | 31 |
| size.ON.concentration.D10 | 1.3986 | 2.3128 | 0.5454 | [-3.1344, 5.9315] | D10 | 31 |
| size.ON.accumulation.D10 | -0.5642 | 2.0539 | 0.7836 | [-4.5898, 3.4614] | D10 | 31 |
| accumulation.ON.concentration.D10 | 1.1105*** | 0.0622 | 0.0000 | [0.9885, 1.2324] | D10 | 31 |
| Variances.elongation.D10 | 0.0617* | 0.0291 | 0.0339 | [0.0047, 0.1188] | D10 | 31 |
| Variances.velocity.D10 | 0.0701* | 0.0331 | 0.0339 | [0.0053, 0.1349] | D10 | 31 |
| Variances.Δcells.D10 | 0.0179* | 0.0084 | 0.0339 | [0.0014, 0.0344] | D10 | 31 |
| Variances.size.D10 | 0.5037* | 0.2374 | 0.0339 | [0.0383, 0.9690] | D10 | 31 |
| Variances.accumulation.D10 | 0.0133* | 0.0063 | 0.0339 | [0.0010, 0.0255] | D10 | 31 |
| Variances.concentration.D10 | 0.3809 | 0.0000 | NA | [0.3809, 0.3809] | D10 | 31 |
| Means.elongation.D10 | -0.0247 | 0.5090 | 0.9613 | [-1.0223, 0.9730] | D10 | 31 |
| Means.velocity.D10 | -0.0466 | 0.5425 | 0.9315 | [-1.1099, 1.0167] | D10 | 31 |
| Means.Δcells.D10 | -0.8382** | 0.2737 | 0.0022 | [-1.3748, -0.3017] | D10 | 31 |
| Means.size.D10 | -0.2152 | 1.4540 | 0.8823 | [-3.0650, 2.6345] | D10 | 31 |
| Means.accumulation.D10 | -0.6926*** | 0.0488 | 0.0000 | [-0.7882, -0.5971] | D10 | 31 |
| Means.concentration.D10 | 0.4832 | 0.0000 | NA | [0.4832, 0.4832] | D10 | 31 |
| elongation.ON.concentration.D16 | -0.9181*** | 0.2277 | 0.0001 | [-1.3644, -0.4718] | D16 | 31 |
| elongation.ON.accumulation.D16 | 0.4262** | 0.1500 | 0.0045 | [0.1322, 0.7202] | D16 | 31 |
| velocity.ON.concentration.D16 | 1.1254 | 0.7202 | 0.1181 | [-0.2862, 2.5369] | D16 | 31 |
| velocity.ON.accumulation.D16 | -0.9541* | 0.4744 | 0.0443 | [-1.8840, -0.0243] | D16 | 31 |
| Δcells.ON.concentration.D16 | -1.1567*** | 0.2406 | 0.0000 | [-1.6282, -0.6853] | D16 | 31 |
| Δcells.ON.accumulation.D16 | 0.6198*** | 0.1585 | 0.0001 | [0.3092, 0.9304] | D16 | 31 |
| size.ON.concentration.D16 | 1.8476*** | 0.5262 | 0.0004 | [0.8163, 2.8790] | D16 | 31 |
| size.ON.accumulation.D16 | -1.3799*** | 0.3466 | 0.0001 | [-2.0593, -0.7005] | D16 | 31 |
| accumulation.ON.concentration.D16 | 1.3570*** | 0.2268 | 0.0000 | [0.9124, 1.8015] | D16 | 31 |
| Variances.elongation.D16 | 0.0357* | 0.0168 | 0.0339 | [0.0027, 0.0687] | D16 | 31 |
| Variances.velocity.D16 | 0.3573* | 0.1684 | 0.0339 | [0.0272, 0.6874] | D16 | 31 |
| Variances.Δcells.D16 | 0.0399* | 0.0188 | 0.0339 | [0.0030, 0.0767] | D16 | 31 |
| Variances.size.D16 | 0.1907* | 0.0899 | 0.0339 | [0.0145, 0.3670] | D16 | 31 |
| Variances.accumulation.D16 | 0.1764* | 0.0832 | 0.0339 | [0.0134, 0.3394] | D16 | 31 |
| Variances.concentration.D16 | 0.3809 | 0.0000 | NA | [0.3809, 0.3809] | D16 | 31 |
| Means.elongation.D16 | 0.2066 | 0.1164 | 0.0759 | [-0.0215, 0.4347] | D16 | 31 |
| Means.velocity.D16 | 0.9398* | 0.3680 | 0.0107 | [0.2185, 1.6612] | D16 | 31 |
| Means.Δcells.D16 | -0.1909 | 0.1229 | 0.1204 | [-0.4319, 0.0500] | D16 | 31 |
| Means.size.D16 | -0.0256 | 0.2689 | 0.9242 | [-0.5526, 0.5015] | D16 | 31 |
| Means.accumulation.D16 | -0.5634** | 0.1778 | 0.0015 | [-0.9118, -0.2149] | D16 | 31 |
| Means.concentration.D16 | 0.4832 | 0.0000 | NA | [0.4832, 0.4832] | D16 | 31 |
| elongation.ON.concentration.D19 | -0.7286** | 0.2484 | 0.0034 | [-1.2156, -0.2417] | D19 | 31 |
| elongation.ON.accumulation.D19 | -0.0288 | 0.1565 | 0.8539 | [-0.3355, 0.2779] | D19 | 31 |
| velocity.ON.concentration.D19 | -0.5829* | 0.2393 | 0.0148 | [-1.0519, -0.1140] | D19 | 31 |
| velocity.ON.accumulation.D19 | 0.0246 | 0.1507 | 0.8701 | [-0.2707, 0.3200] | D19 | 31 |
| Δcells.ON.concentration.D19 | -0.9519* | 0.4568 | 0.0372 | [-1.8472, -0.0566] | D19 | 31 |
| Δcells.ON.accumulation.D19 | 0.2375 | 0.2877 | 0.4090 | [-0.3263, 0.8014] | D19 | 31 |
| size.ON.concentration.D19 | -0.2392 | 0.4217 | 0.5706 | [-1.0657, 0.5873] | D19 | 31 |
| size.ON.accumulation.D19 | 0.1934 | 0.2656 | 0.4664 | [-0.3271, 0.7140] | D19 | 31 |
| accumulation.ON.concentration.D19 | 0.7209 | 0.4716 | 0.1263 | [-0.2033, 1.6451] | D19 | 31 |
| Variances.elongation.D19 | 0.1680* | 0.0792 | 0.0339 | [0.0128, 0.3232] | D19 | 31 |
| Variances.velocity.D19 | 0.1558* | 0.0734 | 0.0339 | [0.0119, 0.2998] | D19 | 31 |
| Variances.Δcells.D19 | 0.5679* | 0.2677 | 0.0339 | [0.0432, 1.0926] | D19 | 31 |
| Variances.size.D19 | 0.4840* | 0.2281 | 0.0339 | [0.0368, 0.9311] | D19 | 31 |
| Variances.accumulation.D19 | 0.7624* | 0.3594 | 0.0339 | [0.0580, 1.4668] | D19 | 31 |
| Variances.concentration.D19 | 0.3809 | 0.0000 | NA | [0.3809, 0.3809] | D19 | 31 |
| Means.elongation.D19 | -0.2023 | 0.2294 | 0.3779 | [-0.6520, 0.2474] | D19 | 31 |
| Means.velocity.D19 | -0.2829 | 0.2210 | 0.2005 | [-0.7160, 0.1502] | D19 | 31 |
| Means.Δcells.D19 | 1.2602** | 0.4219 | 0.0028 | [0.4333, 2.0871] | D19 | 31 |
| Means.size.D19 | 0.5896 | 0.3894 | 0.1301 | [-0.1737, 1.3529] | D19 | 31 |
| Means.accumulation.D19 | -0.9595** | 0.3696 | 0.0094 | [-1.6840, -0.2351] | D19 | 31 |
| Means.concentration.D19 | 0.4832 | 0.0000 | NA | [0.4832, 0.4832] | D19 | 31 |
| elongation.ON.concentration.D21 | -2.6060*** | 0.6146 | 0.0000 | [-3.8106, -1.4013] | D21 | 31 |
| elongation.ON.accumulation.D21 | 1.5826** | 0.5441 | 0.0036 | [0.5161, 2.6490] | D21 | 31 |
| velocity.ON.concentration.D21 | -1.0088 | 0.5292 | 0.0566 | [-2.0460, 0.0285] | D21 | 31 |
| velocity.ON.accumulation.D21 | 0.7615 | 0.4685 | 0.1041 | [-0.1567, 1.6798] | D21 | 31 |
| Δcells.ON.concentration.D21 | -2.6694*** | 0.7649 | 0.0005 | [-4.1686, -1.1701] | D21 | 31 |
| Δcells.ON.accumulation.D21 | 1.2829 | 0.6772 | 0.0582 | [-0.0444, 2.6101] | D21 | 31 |
| size.ON.concentration.D21 | 0.8178 | 0.6515 | 0.2094 | [-0.4591, 2.0947] | D21 | 31 |
| size.ON.accumulation.D21 | -0.2350 | 0.5768 | 0.6836 | [-1.3655, 0.8954] | D21 | 31 |
| accumulation.ON.concentration.D21 | 1.0890*** | 0.1000 | 0.0000 | [0.8931, 1.2850] | D21 | 31 |
| Variances.elongation.D21 | 0.0913* | 0.0430 | 0.0339 | [0.0069, 0.1757] | D21 | 31 |
| Variances.velocity.D21 | 0.0677* | 0.0319 | 0.0339 | [0.0051, 0.1302] | D21 | 31 |
| Variances.Δcells.D21 | 0.1414* | 0.0667 | 0.0339 | [0.0108, 0.2721] | D21 | 31 |
| Variances.size.D21 | 0.1026* | 0.0484 | 0.0339 | [0.0078, 0.1974] | D21 | 31 |
| Variances.accumulation.D21 | 0.0343* | 0.0162 | 0.0339 | [0.0026, 0.0659] | D21 | 31 |
| Variances.concentration.D21 | 0.3809 | 0.0000 | NA | [0.3809, 0.3809] | D21 | 31 |
| Means.elongation.D21 | 1.8797*** | 0.4423 | 0.0000 | [1.0127, 2.7466] | D21 | 31 |
| Means.velocity.D21 | -0.0368 | 0.3808 | 0.9230 | [-0.7832, 0.7097] | D21 | 31 |
| Means.Δcells.D21 | 1.7939** | 0.5505 | 0.0011 | [0.7149, 2.8728] | D21 | 31 |
| Means.size.D21 | -1.0337* | 0.4689 | 0.0275 | [-1.9527, -0.1148] | D21 | 31 |
| Means.accumulation.D21 | -0.7782*** | 0.0784 | 0.0000 | [-0.9318, -0.6246] | D21 | 31 |
| Means.concentration.D21 | 0.4832 | 0.0000 | NA | [0.4832, 0.4832] | D21 | 31 |
| elongation.ON.concentration.D3 | -0.3121 | 0.6976 | 0.6546 | [-1.6793, 1.0551] | D3 | 31 |
| elongation.ON.accumulation.D3 | -0.3354 | 0.6146 | 0.5853 | [-1.5399, 0.8692] | D3 | 31 |
| velocity.ON.concentration.D3 | -2.0609 | 1.7271 | 0.2328 | [-5.4461, 1.3242] | D3 | 31 |
| velocity.ON.accumulation.D3 | 1.2320 | 1.5217 | 0.4182 | [-1.7505, 4.2145] | D3 | 31 |
| Δcells.ON.concentration.D3 | -2.9355** | 1.0651 | 0.0058 | [-5.0230, -0.8480] | D3 | 31 |
| Δcells.ON.accumulation.D3 | 2.1510* | 0.9384 | 0.0219 | [0.3118, 3.9902] | D3 | 31 |
| size.ON.concentration.D3 | 2.2692*** | 0.5343 | 0.0000 | [1.2221, 3.3164] | D3 | 31 |
| size.ON.accumulation.D3 | -1.0392* | 0.4707 | 0.0273 | [-1.9618, -0.1166] | D3 | 31 |
| accumulation.ON.concentration.D3 | 1.1148*** | 0.0753 | 0.0000 | [0.9672, 1.2624] | D3 | 31 |
| Variances.elongation.D3 | 0.0497* | 0.0248 | 0.0455 | [0.0010, 0.0984] | D3 | 31 |
| Variances.velocity.D3 | 0.3046* | 0.1523 | 0.0455 | [0.0061, 0.6031] | D3 | 31 |
| Variances.Δcells.D3 | 0.1158* | 0.0579 | 0.0455 | [0.0023, 0.2293] | D3 | 31 |
| Variances.size.D3 | 0.0291* | 0.0146 | 0.0455 | [0.0006, 0.0577] | D3 | 31 |
| Variances.accumulation.D3 | 0.0164* | 0.0082 | 0.0455 | [0.0003, 0.0326] | D3 | 31 |
| Variances.concentration.D3 | 0.3624 | 0.0000 | NA | [0.3624, 0.3624] | D3 | 31 |
| Means.elongation.D3 | -0.9629*** | 0.2596 | 0.0002 | [-1.4717, -0.4541] | D3 | 31 |
| Means.velocity.D3 | 0.3520 | 0.6428 | 0.5840 | [-0.9078, 1.6118] | D3 | 31 |
| Means.Δcells.D3 | 0.5903 | 0.3964 | 0.1364 | [-0.1866, 1.3672] | D3 | 31 |
| Means.size.D3 | -0.6997*** | 0.1988 | 0.0004 | [-1.0894, -0.3100] | D3 | 31 |
| Means.accumulation.D3 | -0.3935*** | 0.0543 | 0.0000 | [-0.4999, -0.2870] | D3 | 31 |
| Means.concentration.D3 | 0.3975 | 0.0000 | NA | [0.3975, 0.3975] | D3 | 31 |
| elongation.ON.concentration.D8 | -0.7815** | 0.2502 | 0.0018 | [-1.2718, -0.2912] | D8 | 31 |
| elongation.ON.accumulation.D8 | 0.3054* | 0.1264 | 0.0157 | [0.0576, 0.5532] | D8 | 31 |
| velocity.ON.concentration.D8 | 0.9163* | 0.4285 | 0.0325 | [0.0765, 1.7562] | D8 | 31 |
| velocity.ON.accumulation.D8 | -0.5290* | 0.2166 | 0.0146 | [-0.9535, -0.1045] | D8 | 31 |
| Δcells.ON.concentration.D8 | 0.7097 | 0.7961 | 0.3727 | [-0.8507, 2.2701] | D8 | 31 |
| Δcells.ON.accumulation.D8 | -0.5380 | 0.4024 | 0.1812 | [-1.3267, 0.2507] | D8 | 31 |
| size.ON.concentration.D8 | 0.6505* | 0.2953 | 0.0276 | [0.0718, 1.2293] | D8 | 31 |
| size.ON.accumulation.D8 | -0.6693*** | 0.1492 | 0.0000 | [-0.9618, -0.3768] | D8 | 31 |
| accumulation.ON.concentration.D8 | 1.7572*** | 0.3031 | 0.0000 | [1.1631, 2.3512] | D8 | 31 |
| Variances.elongation.D8 | 0.0453* | 0.0214 | 0.0339 | [0.0034, 0.0872] | D8 | 31 |
| Variances.velocity.D8 | 0.1330* | 0.0627 | 0.0339 | [0.0101, 0.2558] | D8 | 31 |
| Variances.Δcells.D8 | 0.4590* | 0.2164 | 0.0339 | [0.0349, 0.8830] | D8 | 31 |
| Variances.size.D8 | 0.0631* | 0.0298 | 0.0339 | [0.0048, 0.1215] | D8 | 31 |
| Variances.accumulation.D8 | 0.3149* | 0.1485 | 0.0339 | [0.0240, 0.6059] | D8 | 31 |
| Variances.concentration.D8 | 0.3809 | 0.0000 | NA | [0.3809, 0.3809] | D8 | 31 |
| Means.elongation.D8 | 1.2124*** | 0.0903 | 0.0000 | [1.0355, 1.3893] | D8 | 31 |
| Means.velocity.D8 | -0.6900*** | 0.1546 | 0.0000 | [-0.9930, -0.3869] | D8 | 31 |
| Means.Δcells.D8 | 0.9222** | 0.2872 | 0.0013 | [0.3592, 1.4852] | D8 | 31 |
| Means.size.D8 | 0.7351*** | 0.1065 | 0.0000 | [0.5262, 0.9439] | D8 | 31 |
| Means.accumulation.D8 | -0.0403 | 0.2376 | 0.8652 | [-0.5060, 0.4253] | D8 | 31 |
| Means.concentration.D8 | 0.4832 | 0.0000 | NA | [0.4832, 0.4832] | D8 | 31 |

*Figure S7: Schematic representation of copper concentration effects on demography, size, elongation, and swimming velocity, and of accumulation level effects on the cited for each strain under the three exposure temperatures (a) 15°C, (b) 23°C and (c) 31°C. Non-significant effects are shown by dotted lines and significant effects by solid lines.*


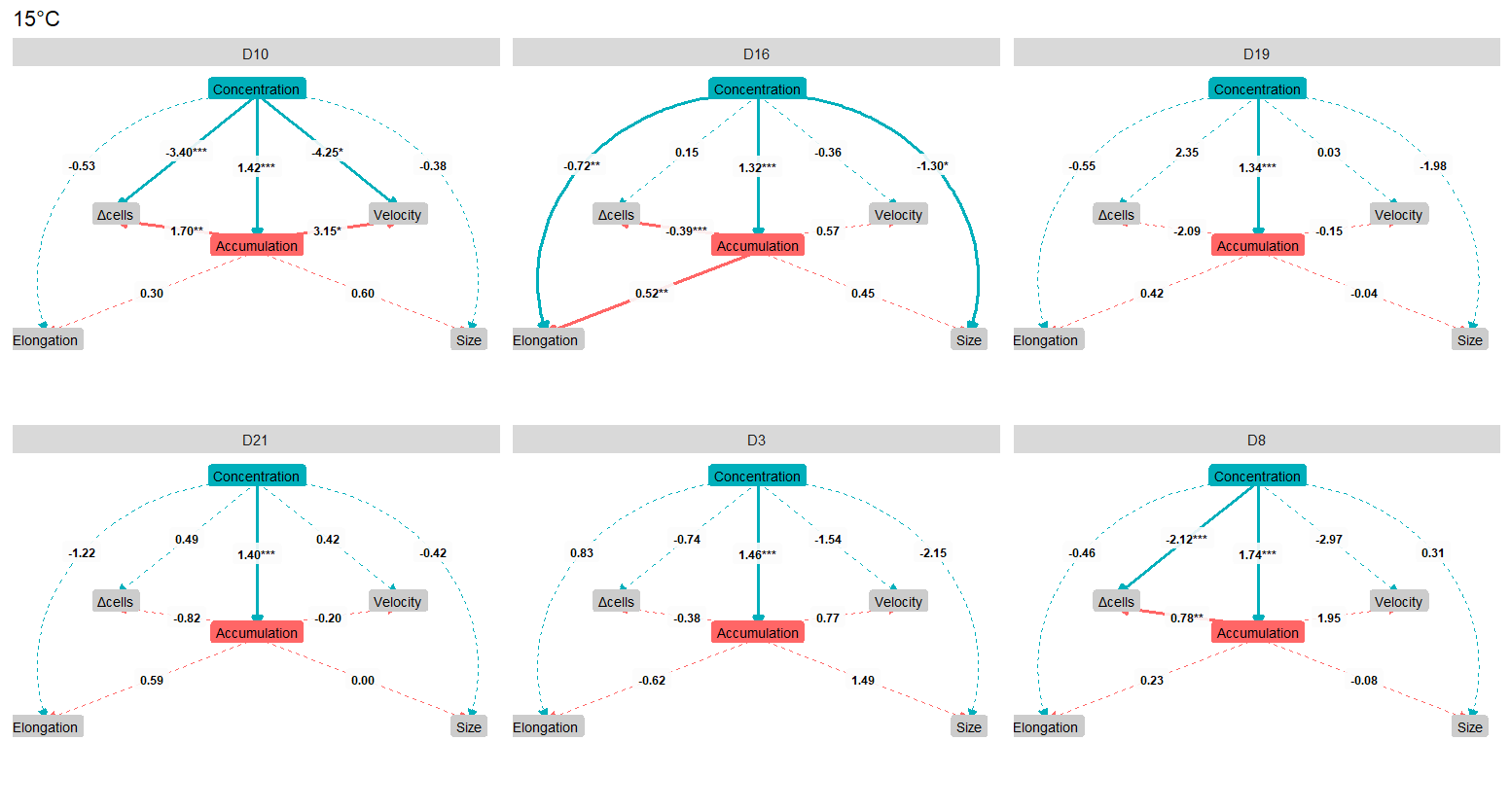


***(a)***

***(b)***


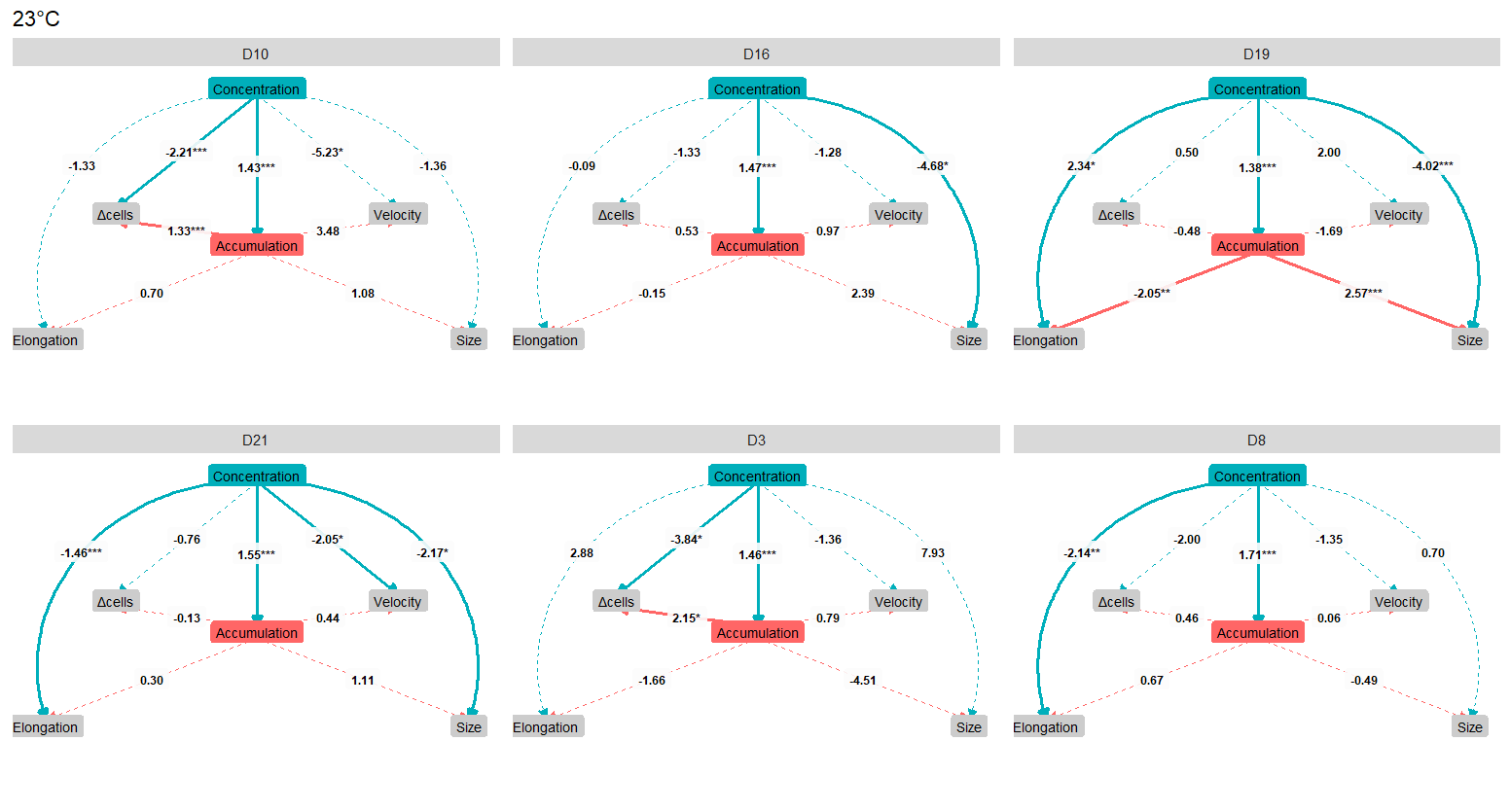


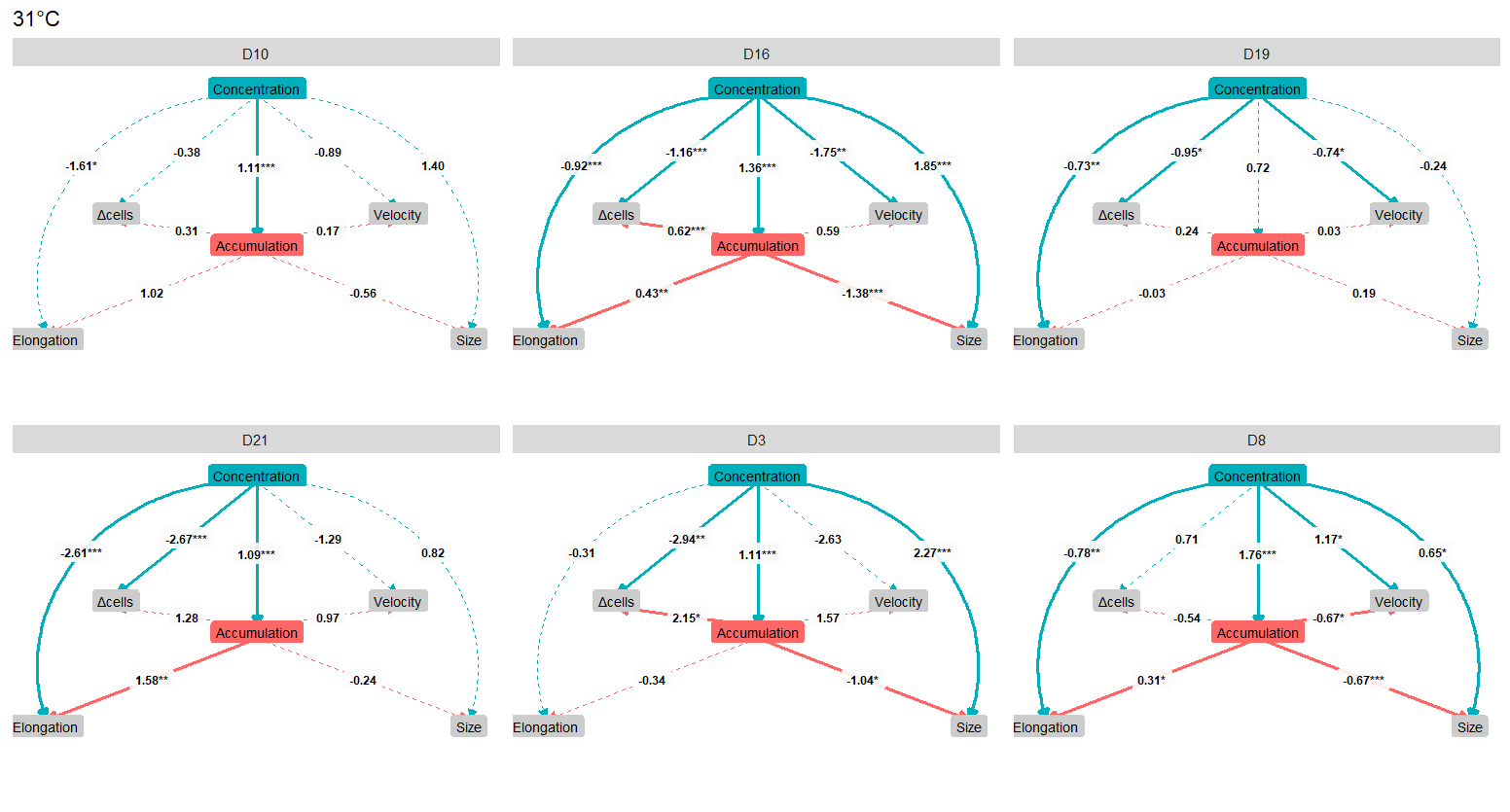


***(c)***

***References***

Jacob, Staffan, Estelle Laurent, Bart Haegeman, Romain Bertrand, Jérôme G. Prunier, Delphine Legrand, Julien Cote, et al. 2018. Habitat Choice Meets Thermal Specialization: Competition with Specialists May Drive Suboptimal Habitat Preferences in Generalists. *Proceedings of the National Academy of Sciences* 115 (47): 11988‑93. <https://doi.org/10.1073/pnas.1805574115>.

Jacob, Staffan, Delphine Legrand. 2021. Phenotypic plasticity can reverse the relative extent of intra- and interspecific variability across a thermal gradient ». *Proceedings of the Royal Society B: Biological Sciences* 288 (1953): 20210428. <https://doi.org/10.1098/rspb.2021.0428>.

Jacob, Staffan, Delphine Legrand, Alexis S. Chaine, Dries Bonte, Nicolas Schtickzelle, Michèle Huet, et Jean Clobert. 2017. « Gene Flow Favours Local Adaptation under Habitat Choice in Ciliate Microcosms ». *Nature Ecology & Evolution* 1 (9): 1407‑10. <https://doi.org/10.1038/s41559-017-0269-5>.

Kahm, Matthias, Guido Hasenbrink, Hella Lichtenberg-Fraté, Jost Ludwig, et Maik Kschischo. 2010. « Grofit: Fitting biological growth curves ». *Nature Precedings* 33 (june). <https://doi.org/10.1038/npre.2010.4508.1>.
